# Successful assisted reproduction treatment and its psychological outcomes for parents and children: a systematic review and meta-analysis

**DOI:** 10.1007/s10815-025-03572-9

**Published:** 2025-07-09

**Authors:** Julia Jeannine Schmid, Ulrike Ehlert

**Affiliations:** 1 Department of Clinical Psychology and Psychotherapy, University of Zurich, Zurich, Switzerland; 2 URPP Human Reproduction Reloaded, University of Zurich, Zurich, Switzerland

**Keywords:** Assisted reproduction, Stress, Mental health, Parenting, Child development, Meta-analysis

## Abstract

**Purpose:**

Overcoming infertility through assisted reproductive technology (ART) is stressful and requires a high level of investment. Infertility, ART, and associated stress may continue to affect the family even after birth. This systematic review and meta-analysis compared psychological outcomes in families formed through ART (ART families) and families formed through natural conception (NC families), while examining factors that may promote healthy development in ART families.

**Methods:**

We systematically searched PsycINFO, PsycArticles, and PubMed for studies published up to mid-October 2024 comparing aspects of psychological family functioning in ART and NC families beyond the first year postpartum. Eighty-nine studies were included in the review and 33 in the random-effects meta-analysis.

**Results:**

Mental health and parental relationship quality were comparable between ART and NC parents. Compared to NC mothers, ART mothers reported slightly lower parenting stress, comparable to better mother-child relationships, and higher parental commitment. ART and NC children showed comparable intelligence and cognitive and psychomotor development, with evidence of better language skills and slightly lower school performance in ART children. Psychosocial development was marginally better in ART children according to mothers’ reports, although mental health outcomes were comparable or slightly worse.

**Conclusion:**

Overall, family functioning after ART seems to be similar to that of NC families. ART parents may exhibit protective sociodemographic characteristics, high resilience, and strong maternal commitment, potentially buffering infertility, ART, and stress effects. Further investigation is warranted to address methodological limitations evident in the existing literature and to explore protective factors in ART families.

**Graphical Abstract: Comparison of psychological outcomes in mothers, fathers, and children after assisted reproduction and natural conception:**

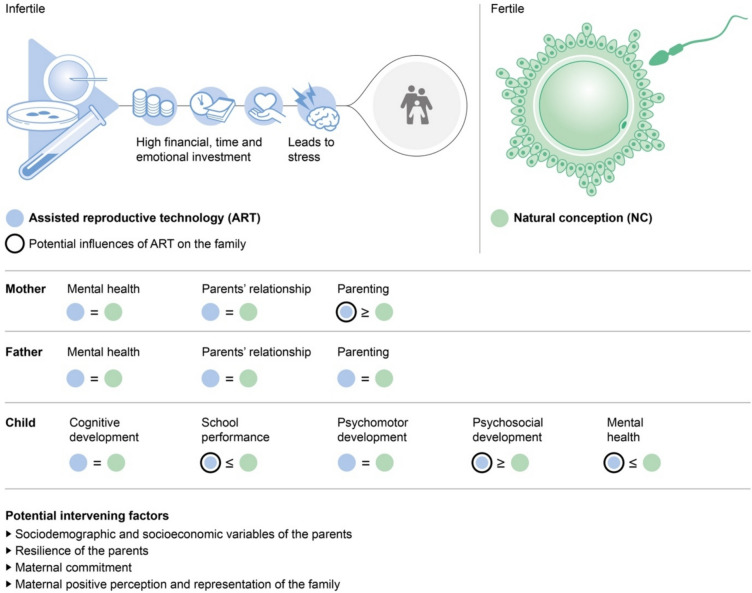

**Supplementary Information:**

The online version contains supplementary material available at 10.1007/s10815-025-03572-9.

## Introduction

Infertility is a global public health problem affecting one in six people worldwide [1]. It impacts the mental health of the couples concerned, their social relationships, and romantic partnership [2]. Many of those affected report stress, sadness, anxiety, and depressed mood [2–4].

In today’s world, however, infertility is no longer something that simply has to be accepted. More and more couples are seeking medical help in the form of assisted reproductive technology (ART) to increase their chances of parenthood despite infertility [5]. ART is defined as fertility treatments in which eggs are treated outside the body, like in vitro fertilization (IVF) and intracytoplasmic sperm injection (ICSI) [6]. Worldwide, about 4 million ART treatments are performed annually, and 1000 000 children are born [7].

The use of ART is associated with physical and psychological stress, requiring a great deal of financial, time, and emotional investment [8, 9]. Stressors include stigmatization, high financial costs, medical procedures, high uncertainty due to a low success rate, long waiting times, the emotional rollercoaster of hope and disappointment, failed treatment attempts, and concerns about an increased risk of complications during pregnancy [4, 10–15]. Even during pregnancy, women who conceived through ART show higher stress levels [e.g. 8, 16, 17]. In sum, achieving parenthood through ART requires greater effort than parenthood through natural conception (NC).

The question of whether infertility, ART, and the associated burdens continue to affect the family after childbirth is becoming ever more relevant with the increasing use and success rate of ART. While most studies addressing this question have focused on medical outcomes, some psychological studies have examined parental mental health, parenting, and child development after successful ART.

**Fig. 1:**
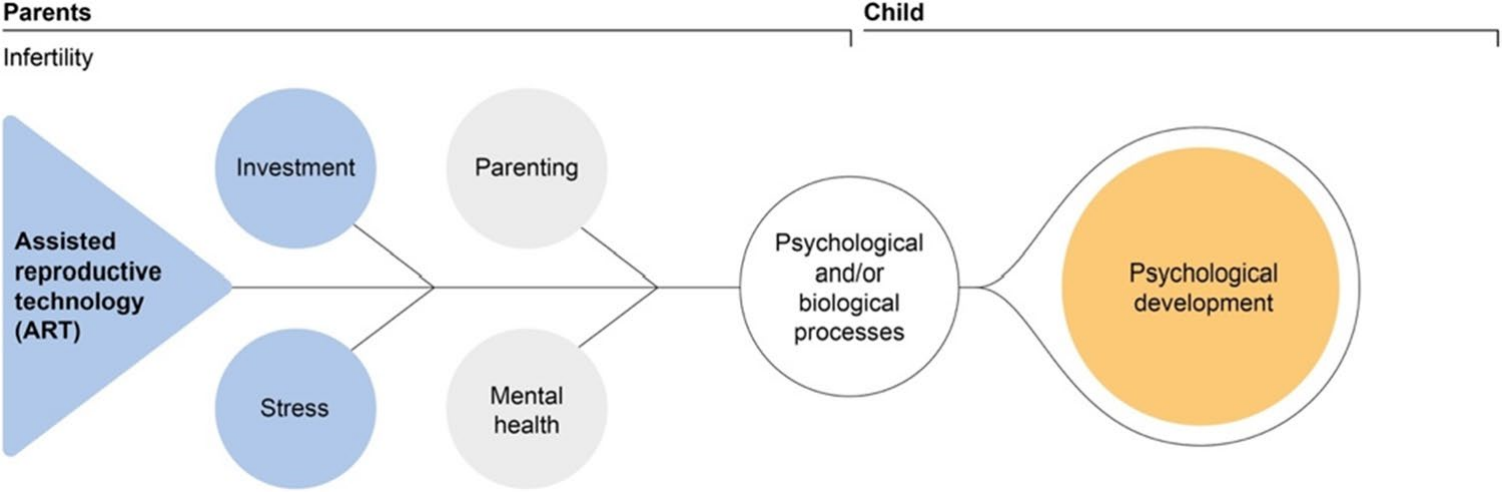
Potential mechanisms of influence of ART on the family

Many of these psychological studies share the assumption that infertility and ART itself, the associated stress, or the effort required to become a parent might affect the parents and the offspring. Such effects may be related to biological and psychological processes (Fig. 1). Specifically, the high levels of effort and stress may affect ART couples’ mental health during treatment and pregnancy [18]. This influence on parental mental health might persist after childbirth [19–22], with a potential bidirectional association between parental mental health and parenting [e.g. 23]. Simultaneously, maternal stress may impair child development through biological processes (hormonal and epigenetic changes) [17, 24–28], and the associated parenting behavior, such as overprotection, may further affect child development [29–31]. From a medical perspective, infertility and/or ART might lead to biological changes (e.g. epigenetic changes), which may likewise affect the child’s development [32–36]. For example, ART children have an increased risk of preterm birth and low birth weight, mainly due to the increased incidence of twin pregnancies, and these factors are in turn associated with poorer child development [37–40].

While research conducted to date provides some indications of the individual correlations, the complete model is yet to be tested. Interestingly, the available reviews regarding psychological family functioning after successful assisted reproduction suggest that families formed through ART (ART families) do not differ greatly from families formed through natural conception (NC families) in terms of maternal mental health [e.g. 41], parenting [e.g. 42, 43, 44] and the children’s cognitive and psychosocial development [e.g. 45–47] (for a summary, see Supplement 1). However, they do highlight the need for high-quality studies that capture long-term effects and consider covariates (e.g. preterm birth). While these previous reviews offer valuable insights into the complex area of family functioning after ART, most are outdated, child-centered, narrative, and lack meta-analyses (Supplement 1). Moreover, some have a medical rather than psychological focus and some do not consistently distinguish between different infertility treatments. Given that ART is the most effective, yet stressful, infertility treatment [29, 48], a focus on ART would be illuminating from both a medical and psychological perspective.

## Methods

### Search strategy

To identify relevant papers, we conducted a systematic search using the electronic databases PsycINFO, PsycArticles, and PubMed up to mid-October 2024 (Table 1). Search terms included keywords related to ART (e.g. “infertility treatment*”) and family members (e.g. parent*). The search yielded 5,530 peer-reviewed articles.

**Table 1:** Search strategy used to identify studies for inclusion

Databases	Keywords	Restrictions	Number of articles
PubMed	Group 1 (“infertility treatment*” OR “fertility treatment*” OR “reproductive technolog*” OR “reproductive techniques” OR “artificial reproduction*” OR “assisted reproduction*” OR “assisted conception*” OR “artificial conception*” OR “in vitro fertilisation*” OR “in vitro fertilization*” OR “IVF” OR “intracytoplasmic sperm injection*” OR “ICSI” OR “embryo transfer*”) AND Group 2 (parent* OR mother* OR father* OR maternal OR paternal OR parental OR maternity OR paternity OR parenting OR family OR families OR infant* OR child* OR kid*) AND Group 3 (“mental health” OR stress* OR distress OR resilience OR vulnerab* OR “affective disorder*” OR anxiet* OR depression* OR psych* OR emotional* OR well-being OR wellbeing OR “life satisfaction” OR “life quality” OR “quality of life” OR coping OR “social behavior” OR socioemotional OR partnership OR relationship OR interaction OR adaptation OR adjustment)	Group 1 had to appear in the title Group 2 had to appear in the title or the abstract English articles only Only studies conducted in humans	3,303
PsycINFO	Group 1 AND Group 2	Excluding dissertations and books	2,167
PsycArticles	Group 1 AND Group 2	No restrictions	60

### Study selection

**Fig. 2:**
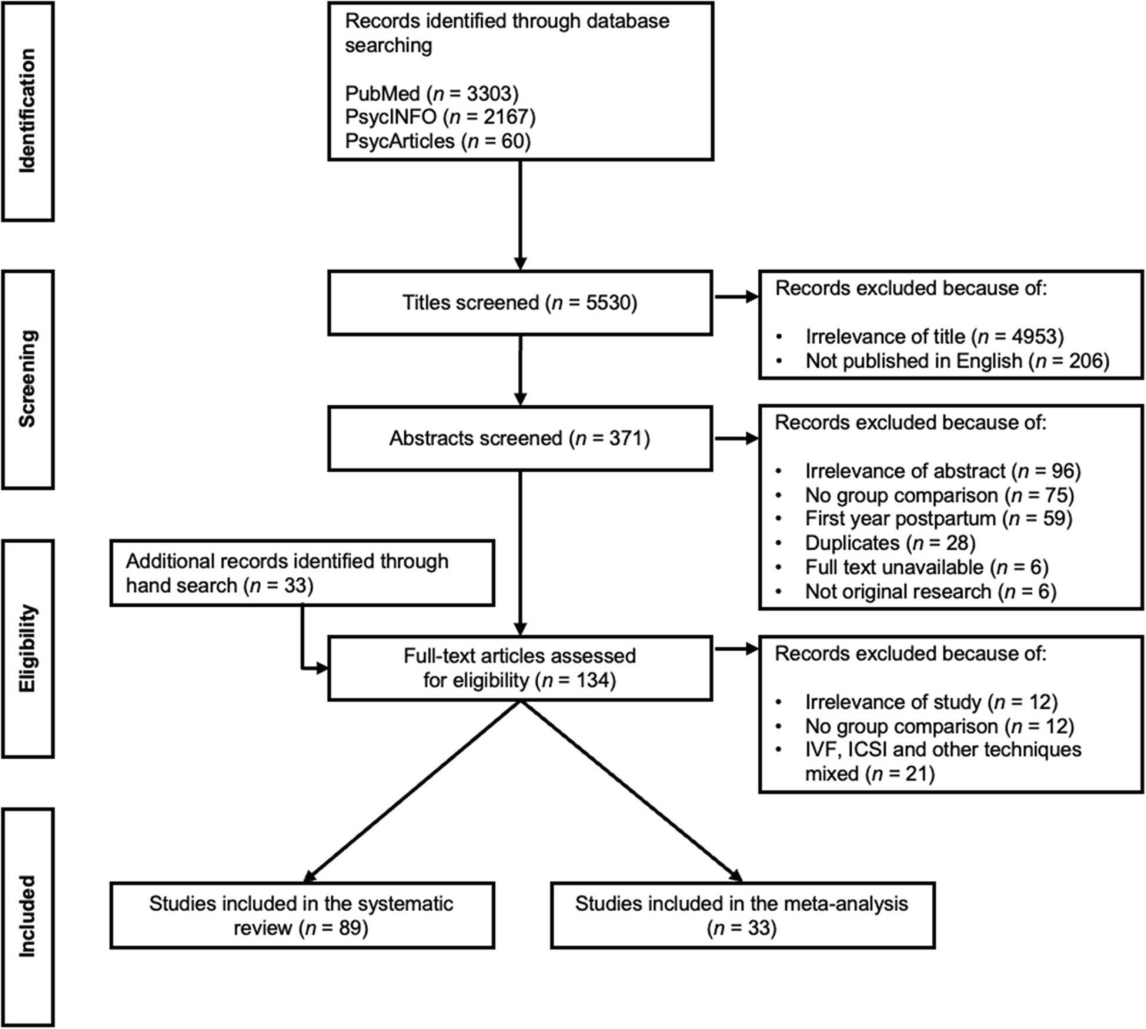
Flowchart of the systematic literature search and study selection process

The titles and abstracts of the identified papers were screened to check the inclusion and exclusion criteria. Studies were included if they a) assessed the psychological functioning of the family or individual family members after successful ART, and b) compared these families with families formed by NC c) beyond the first year postpartum. Successful ART was defined as IVF or ICSI resulting in childbirth. We did not include studies that examined other infertility treatments, such as intrauterine insemination and ovulation induction, or studies in which participants receiving ART and participants receiving other treatments were mixed within one study group [e.g. 50]. Information on conception method was obtained from different sources across the studies, including self-reports, hospital records, and registry data. Psychological functioning encompassed parents’ mental health, parenting, and the children’s psychological development. We considered only English-language papers for which the full text was available. After removing duplicates, we read the full texts of the remaining articles to double-check the criteria. The reference lists of these papers were hand-searched for further relevant articles, resulting in the inclusion of 33 additional articles (Fig. 2).

### Data extraction and quality assessment

The studies selected were tabulated in an Excel document for data extraction. We extracted information on the first author, year of publication, country, study design, sample, assessment time points, outcome measures, and main results (group differences). If a paper did not report whether ART and NC families significantly differed on an outcome of interest, we calculated the significance using a t-test based on the available values (e.g. means, standard deviations).

Risk of bias was evaluated using a quality rating scale (Supplement 2) adapted from previous meta-analyses [51–53]. This scale was chosen as it could be easily adapted to the research area, enabling specific methodological challenges to be assessed (e.g. past infertility in the control group, consideration of specific covariates). All items were rated on the original three-point scale (0–2), with a maximum possible quality score of 16 (100%). The quality of the studies was independently assessed by one of the study investigators and a research assistant. Subsequent descriptive analyses were conducted to determine whether the direction of the results was related to the study quality, the control for key covariates (family size, multiple births, parental age, parental SES (operationalized differently across studies), preterm birth/birth weight), and the time point at which the family was studied. As most of the studies that were potentially conducted during the COVID-19 period did not report whether data collection occurred during the pandemic, it was not possible to control for this factor in the analyses (e.g. impact on mental health of the family members).

### Data analysis

To investigate the differences between ART and NC families, we conducted random-effects meta-analyses using SPSS, as we expected true effect sizes to vary across studies due to methodological and sample-related differences (e.g. child age, exclusion criteria) [54]. If additional data were needed, we attempted to contact the respective study authors. Studies with insufficient data were only included in the systematic review. A meta-analysis was computed if at least two studies with non-overlapping samples used the same measure. In the case of overlapping samples, the study with the largest sample size was included. Reasons for excluding studies from meta-analyses are provided in Supplement 3 for each study. We used mean values and standard deviations to calculate Hedges’ *g*. When values from two combined groups were required (e.g. ICSI and IVF groups), the combined mean and combined standard deviation were determined. To assess the heterogeneity of the included studies, *I*^*2*^ statistics were calculated and the study designs were compared. As there were fewer than 10 studies per meta-analysis, no additional statistical method could be used to analyze publication bias and sensitivity. Meta-analytic results were considered robust if all samples could be included, or if the results yielded the same conclusion as the systematic review and heterogeneity was at most 40% [55].

## Results

### General study characteristics

The study selection is summarized in Fig. 2 and the study characteristics are presented in Supplement 3. In total, 89 publications were included in the systematic review and were divided into three categories: possible impact on the parents (*n* = 13), on parenting (*n* = 22), or on the child (*n *= 80). Some reports were categorized into more than one group. The studies were published between 1990 and 2024. They were mainly conducted in Europe and North America, particularly in the United Kingdom, the Netherlands, Sweden, and the United States. The sample sizes ranged from 26 to many thousands of participants. The mean age of the children in the studies ranged from one to 28 years.

The interrater reliability of the quality rating was calculated using Krippendorff’s alpha, and showed very good agreement (*α* = 0.91) [56]. The studies scored an average of 47% in the quality rating (Range 13–81%). Notably, as all studies are observational, they do not allow for causal conclusions. Table 2 contains the number and quality of studies per category. In the following, the results of the 89 studies are summarized according to their outcome variable. The results of the meta-analyses are presented in Table 3. Table 4 presents the final results of the comparison of ART and NC families, taking into account the systematic review and the meta-analyses.

**Table 2:** Number and quality of studies per category

Variable	Number of studies	Number of studies with sample overlap	Mean QR in %
**Possible impact of successful ART on the parents**			
Mental health (m)	9	3	43%
Mental health (f)	10	6	42%
Parents’ relationship	11	4	42%
**Possible impact of successful ART on parenting**			
Parenting stress	12	4	44%
Parent–child relationship	11	6	44%
Parenting behavior	15	8	43%
**Possible impact of successful ART on the child**			
Cognitive development	41	21	49%
Psychomotor development	21	5	49%
Psychosocial development and mental health	55	31	44%

f, father; m, mother; NC, natural conception; QR, quality rating

**Table 3:** Meta-analyses of the comparison of ART and NC families

Variable		Measurement instrument	Number of studies	Mean QR in %	*g*	95% CI	Z	*p*	*I*^*2*^
**Possible impact of successful ART on the parents**									
Mental health (m)	Depression	BDI ^a^	2	40%	0.13	−1.04, 1.28	0.21	0.833	92%
	Anxiety	STAI ^a^	3	48%	0.14	−0.77, 1.06	0.31	0.758	91%
Mental health (f)	Anxiety	STAI ^b^	2	54%	−0.60	−0.97, −0.23	−3.18	0.001**	0%
**Possible impact of successful ART on parenting**									
Parenting stress		PSI (m) ^b^	5	51%	−0.17	−0.29, −0.04	−2.50	0.012*	23%
		PSI (f)	4	48%	−0.12	−0.27, 0.01	−1.73	0.080	0%
**Possible impact of successful ART on the child**									
Cognitive development	IQ	Kaufman scales ^a^	4	44%	−0.05	−0.23, 0.14	−0.49	0.627	57%
		Wechsler scales ^a^	6	56%	−0.05	−0.33, 0.23	−0.32	0.747	87%
	Cognitive ability	Vineland – C	2	44%	0.48	0.16, 0.80	2.96	0.003**	18%
Psychomotor development	Visual-motor function	Beery – VMI	2	47%	0.03	−0.14, 0.19	0.32	0.746	0%
		Beery – VP	2	47%	−0.17	−0.37, 0.02	−1.79	0.074	27%
	Overall psychomotor development	Vineland – MS ^a^	2	44%	0.66	−0.08, 1.39	1.74	0.081	83%
		BLS ^a^	2	75%	−0.00	−0.33, 0.32	−0.01	0.992	64%
Psychosocial development and mental health	Psychosocial development	CBCL	9	42%	−0.08	−0.17, 0.01	−1.70	0.089	40%
		YSR	3	45%	−0.03	−0.17, 0.11	−0.45	0.656	0%
		TRF	3	62%	−0.07	−0.28, 0.14	−0.65	0.513	0%
		SDQ	4	36%	−0.14	−0.20, −0.07	−4.25	< 0.001***	0%
		Vineland – S	2	41%	0.40	0.06, 0.75	2.30	0.022*	29%
		ECBI	2	54%	−0.33	−0.54, −0.13	−3.17	0.002**	0%

^a^ Due to substantial heterogeneity (> 40%), the results should be interpreted with caution. ^b^ Results should be interpreted with caution, as it is unclear whether the detected effects would persist if all studies were included. *p < 0.05, **p < 0.01, ***p < 0.001. 95% CI, 95% confidence interval; BDI, Beck Depression Inventory [57]; Beery VMI/VP, Beery-Buktenica Developmental Test of Visual-Motor Integration/Visual Perception [190–192]; BLS, Brunet-Lézine Scale [193, 194]; CBCL, Child Behavior Checklist [131]; ECBI, Eyberg Child Behavior Inventory [145]; f, father; g, effect size Hedges’ g; I^2^, heterogeneity; Kaufman scales, Kaufman Assessment Battery for Children (K-ABC), Kaufman Brief Intelligence Test (K-BIT-2) [86, 87]; m, mother; NC, natural conception; PSI, Parenting Stress Index [75]; QR, quality rating; SDQ, Strengths and Difficulties Questionnaire [139, 140]; STAI, State-Trait Anxiety Inventory [64, 65]; TRF, Teacher’s Report Form [132]; Vineland – C/MS/S, Vineland Adaptive Behavior Scales – Communication/Motor skills/Socialization [147, 148]; Wechsler scales, Wechsler Abbreviated Scale of Intelligence (WASI), Wechsler Intelligence Scale for Children – Revised (WISC-R), Wechsler Preschool and Primary Scales of Intelligence – Revised (WPPSI-R) [92–94]; YSR, Youth Self-Report [133]; Z, Test statistics Z

**Table 4:** Summary of the comparison of ART and NC families

Variables	Mother	Father	Child
Parents’ mental health	Comparable	Comparable	^a^
Parents’ relationship	Comparable	Comparable	^a^
Parenting stress	Lower to comparable	Comparable	^a^
Parent–child relationship	Comparable to better	Comparable	Comparable
Parenting behavior	Comparable parenting style Indication of higher parental commitment	Comparable	Comparable
Child cognitive development	^a^	^a^	Comparable IQ and cognitive development Evidence of better language development Lower to comparable school performance
Child psychomotor development	^a^	^a^	Comparable
Child psychosocial development and mental health	^a^	^a^	Comparable to better psychosocial development ^b^ Poorer to comparable mental health

Table represents the final results, taking into account the systematic review and the meta-analytic data. ^a^ Not applicable. ^b^ Slightly better psychosocial development in ART than NC children according to mothers’ reports. NC, natural conception.

### Possible impact of successful ART on the parents

Regarding *maternal mental health,* six studies assessed *depressive symptoms* in ART and NC mothers, four using the Beck Depression Inventory (BDI) [57]. Of these, one study reported lower depressive symptoms in IVF mothers than in NC mothers 4–8 years postpartum [58], but follow-ups of these families revealed that symptoms were comparable between the two groups at 11–12 [59] and 17–19 years postpartum [60]. Conversely, another study reported higher BDI scores in ART mothers than in NC mothers 2–13 years postpartum [61]. The two studies using other measures found no significant differences [62, 63]. The meta-analysis yielded a null finding (number of studies (k) = 2, *p* = 0.833).

*Anxiety symptoms* in ART and NC mothers were examined in six studies, using the State-Trait Anxiety Inventory (STAI) [64, 65]. One study reported lower trait anxiety in IVF mothers than in NC mothers 4–8 years postpartum [58], but anxiety levels were similar at 11–12 [59] and 17–19 years postpartum [60]. Two studies reported comparable anxiety levels up to five years after delivery [63, 66]. The sixth study found higher state and trait anxiety in ART mothers 2–13 years postpartum [61]. The meta-analysis revealed no significant differences (k = 3, *p* = 0.758).

Two studies investigated the *general mental health* of ART and NC mothers using the General Health Questionnaire (GHQ-28) [67] and found no group differences approximately five years postpartum [66, 68]. Kiesswetter et al. [69] reported similar life satisfaction, stress, and worry 1.5 years postpartum, but lower worry in IVF than NC mothers two years postpartum.

Regarding *paternal mental health,* five studies assessed *depressive symptoms* in IVF and NC fathers, four using the BDI; all showed comparable levels [58–60, 63, 70].

*Anxiety symptoms* in IVF and NC fathers were assessed in six studies, using the STAI, of which one reported lower anxiety levels in IVF fathers [58] whereas the others found similar levels [59, 60, 63, 66, 70]. The meta-analysis revealed lower trait anxiety in IVF fathers, although only two studies could be included (k = 2, *p* = 0.001**).

Two studies evaluated the *general mental health* of ART and NC fathers using the GHQ-28 approximately five years postpartum, finding similar total scores [66, 68]. In the study by Golombok et al. [58], IVF fathers were less likely to have sought psychiatric treatment. Kiesswetter et al. [69] observed similar life satisfaction, stress, and worry 1.5 years postpartum, but lower worry in IVF than NC fathers two years postpartum. Taubman-Ben-Ari et al. [71, 72] reported comparable life satisfaction, posttraumatic growth, and positive and negative affect in both groups.

Looking at the *couple’s relationship,* Sydsjö et al. [73] revealed a more stable relationship in IVF parents than in NC parents during the first five years postpartum, while all other studies indicated comparable relationship quality [19, 58–60, 63, 66, 68, 70, 71, 74].

### Possible impact of successful ART on parenting

Eleven studies assessed *parenting stress* using the Parenting Stress Index (PSI) [75]. Six studies found comparable levels between ART and NC mothers and seven found comparable levels between ART and NC fathers [19, 58, 66, 68, 70, 72, 76–78]. However, McMahon et al. [66] noted that higher levels of IVF treatment were associated with less parenting stress, and Hahn and DiPietro [19] found that IVF mothers with only one child reported less parenting stress compared to IVF mothers with more than one child and NC mothers. The results of Ponjaert-Kristoffersen et al. [79] and van Balen [76] suggested less parenting stress in ART mothers than in NC mothers, while Golombok et al. [58] reported less parental distress in IVF fathers than NC fathers. In contrast, Knoester et al. [80] found higher parenting stress in ICSI parents than in NC parents due to child characteristics, and Cook et al. [81] reported higher parenting stress in parents of IVF twins than in parents of NC twins. The meta-analysis showed lower parenting stress in ART mothers than in NC mothers (k = 5, *p* = 0.012*) and comparable levels among fathers (k = 4, *p* = 0.080).

The* parent–child relationship* was assessed, for example, by Van Balen [76], who reported that IVF mothers of 2–4-year-olds experienced more pleasure and expressed stronger feelings towards their child compared to NC mothers; no significant differences were found between fathers. Barnes et al. [68] found fewer aggressive or hostile feelings in ICSI mothers towards their five-year-old children than in NC mothers, while the father-child relationship did not differ. Golombok et al. [58] reported greater interaction with 4–8-year-old children in IVF parents than in NC parents, although this difference disappeared by age 12 [82]. In some but not all of their studies, Golombok’s research group also found differences in conflict behavior between IVF and NC families, such as greater disciplinary indulgence in IVF mothers than NC mothers [59, 60, 82]. Other studies suggested comparable parent–child relationships among ART and NC families [59, 62, 63, 82, 83]. Children’s reports revealed no differences in parent–child attachment and in affection and feelings towards their parents [58, 59, 68, 70, 82, 84].

Previous studies have investigated *parenting behavior* in general and individual aspects thereof, particularly involvement in parenthood, which is also referred to as parental commitment (though the latter carries a slightly more positive connotation) and means the emotional, cognitive, and behavioral investment in the parenting role, including both positive and negative aspects, such as concerns about the child and prioritizing the child's needs (cf. [58, 68]). Colpin’s research group found no effect of conception mode on parenting style as reported by fathers, mothers, and children at 8–9 or 15–16 years postpartum [77, 78]. Conversely, Golombok’s research group [58, 59, 82] noted some differences, including higher emotional involvement in IVF than NC parents. These differences disappeared by 18–19 years postpartum [60, 84] and did not exist for mothers of twins [81]. Regarding parenting goals, IVF and NC parents showed many similarities, but certain differences emerged; for example, IVF fathers prioritized child adjustment to expectations more than did NC fathers [74, 76, 77].

In terms of involvement in parenthood, Egan et al. [21] suggested that ART mothers were more likely to perceive their child as vulnerable, especially when donor gametes were used. Hahn and DiPietro [19] reported that IVF mothers were more protective, had higher separation anxiety, and were more likely to encourage dependency than NC mothers, though teachers saw IVF mothers as more openly affectionate but not overprotective towards their children. Further studies suggested no group differences based on conception mode regarding parenting anxiety [74], parental concerns [76], and parents’ emotions about separation from their 18-year-old offspring [60]. However, Barnes et al. [68] reported that ICSI parents showed the highest parental commitment, followed by IVF and NC parents, with significant differences between ICSI and NC mothers. Carson et al. [83] found that more ART mothers read daily to their children, and Hahn and DiPietro [19] reported that IVF mothers were more likely to provide their children with preschool enrichment programs compared to NC mothers.

Findings on family functioning after ART were mixed: IVF mothers were less satisfied with aspects of family functioning compared to NC mothers [19], whereas ART parents reported less emotional burden from having a child and of the child impacting negatively on family life [74]. Fisher et al. [85] showed that ART mothers were over three times more likely to use residential early parenting services within 18 months postpartum than the general population.

### Possible impact of successful ART on the child

Regarding *cognitive development*, studies have compared IQ, cognitive abilities, and school performance between ART and NC children. Four studies using Kaufman intelligence tests [86, 87] found no *IQ* differences between 4–9-year-old ART and NC children, even after accounting for covariates [88–91]. However, Schendelaar et al. [90] noted a negative correlation between time to conceive and IQ. Nine studies using Wechsler scales [92–94] mostly reported comparable IQ among 3–10-year-old ART and NC children, at least after controlling for covariates [79, 95–101]. In contrast, Leunens et al. [102] found slightly higher IQ in 8-year-old ICSI children, possibly attributable to higher maternal education, but this difference disappeared by age ten [99]. Results from other IQ tests were inconsistent. Compared to NC children, Pottinger and Palmer [74] reported higher IQ in IVF children aged ≤ 7 years, Liapi and Polychronopoulou [103] found similar IQ in 8–10-year-old ICSI and NC children, and Knoester et al. [104] found lower IQ in 5–8-year-old ICSI children. In Knoester et al. [104], the effect size depended on the control variables included, e.g. parental age increased it. Meta-analyses revealed no difference between ART and NC children (Kaufman intelligence tests: k = 4, *p* = 0.627; Wechsler scales: k = 6, *p* = 0.747).

*Cognitive abilities* of ART and NC children were examined in eighteen studies, with differences often attributed to parental variables (e.g. age, education). For example, Barbuscia and Mills [105] found higher verbal cognitive abilities in ART than NC children at ages three and five, but this difference diminished by age 11. The difference persisted after controlling for multiple births, low birth weight, and parity, but disappeared when controls for parental variables were added. Carson et al. [83, 106] reported similar spatial and non-verbal abilities, but higher verbal ability in ART than NC children at ages three and five, explained by parental characteristics. Interestingly, controlling for gestational age and birthweight increased the difference, whereas controlling for parental involvement (e.g. frequency of reading to the child) slightly reduced it [83, 106]. Guo et al. [101] found no mean differences in cognition measures, but fewer IVF than NC children had below-average cognitive development, which was associated with maternal education. Farhi et al. [91] reported similar language skills among 7–9-year-old ART and NC children after adjusting for parental variables, though ART children had fewer disabilities in dictation initially. Aoki et al. [107, 108] found no significant difference between ART children and NC children at ages two and five, but at age three, ART children showed better language development when controlling for parental variables and multiple births and at age four, ART boys showed better receptive language development than NC boys. Two further studies likewise reported better communication skills in ART than NC children after controlling for parental variables [74, 109]. In the remaining studies, no differences in cognitive abilities between ART and NC children were observed after adjustment for covariates [95, 110–114]. However, most studies did not specify whether differences existed without these adjustments. Cederblad et al. [115] did not adjust for parental variables, and found similar cognitive development in IVF and NC children. The prevalence of mental retardation or developmental disorders was similar [116] or slightly increased in IVF compared to NC children [117]. Neuropsychological development [100, 112] and the executive function [91, 113] were comparable between ART and NC children. The meta-analysis suggested better communication skills in ART children compared to NC children (Vineland – Communication: k = 2, *p* = 0.003**).

Fifteen studies analyzed the *school performance* of ART and NC children. Whether differences were found seemed to depend largely on the covariates used. For example, Mains et al. [118] and Luke et al. [119, 120] stated that the better school performance of IVF children in their studies was probably due to insufficient control for parental variables. Additionally, six studies revealed that ART children showed better school performance before controlling for parental variables, but comparable or worse performance after adjustment [121–126]. In contrast, Levy-Shiff et al. [95] found no differences in reading comprehension and learning adjustment for 9–10-year-old matched IVF and NC children. Wagenaar et al. [111], Liapi and Polychronopoulou [103], Al-Hathlol et al. [127], and Kennedy et al. [128] found no difference between IVF and NC groups regarding performance in different educational domains, the numbers of children who had to repeat school years, learning difficulties, extra lessons, or special educational needs, even after adjusting for covariates (e.g. maternal education, gestational age). However, Al-Hathlol et al. [127] noted that more IVF children had received preschool education compared to NC children.

Several studies have focused on *psychomotor development* of ART children, investigating gross motor function, fine motor function, visual-motor function, and overall psychomotor development. Eight studies, encompassing children aged 2–16 years, investigated *gross motor function*, and indicated comparable development between ART and NC children [79, 88, 99, 101, 102, 107, 108, 127]. Parental variables did not seem to influence these findings [79, 88, 107, 127].

Six studies suggested comparable *fine motor function* in ART and NC children [79, 101, 107–109, 113].

The *visual-motor function* of ART and NC children also appeared to be comparable [91, 95, 113]. Wagenaar et al. [112] found slightly lower visual-motor function in the IVF group, but scores were within the normal range. Regarding the Beery-Buktenica test, the meta-analysis yielded non-significant findings (Visual Motor Integration: k = 2, *p* = 0.746; Visual Perception: k = 2, *p* = 0.074).

*Overall psychomotor development* was assessed in eleven studies, including children up to age 17 years. Six of these indicated no differences between ART and NC children [62, 88, 97, 98, 116, 129]. However, Pottinger and Palmer [74] and Friedlander et al. [109] reported better motor abilities in ART children than in NC children aged ≤ 7 years. Guo et al. [101] found no mean differences in psychomotor development, but reported that fewer IVF than NC children had below-average development. The study by Kelly-Vance et al. [110] revealed lower psychomotor development in 2-year-old ART twins compared to NC twins, and Vo et al. [130] reported some evidence of slower language and motor coordination development in ICSI children up to age 2.5 years compared to their NC counterparts. The meta-analysis yielded non-significant findings regarding the Vineland Adaptive Behavior Scales II (k = 2, *p* = 0.081) and the Brunet-Lezine scale (k = 2, *p* = 0.992).

*Psychosocial development* was mostly assessed using Achenbach’s questionnaires [131–133], yielding mixed results. Twelve studies showed comparable Child Behavior Checklist (CBCL), Youth Self-Report (YSR), or Teacher’s Report Form (TRF) total scores and comparable prevalences for disturbed behavior between ART and NC children [61, 68, 77, 78, 80, 90, 100, 103, 115, 134–136]. Özbaran et al. [61] found higher internalizing and externalizing scores in ART children. In the study by Wijs et al. [136], ART children showed fewer self-reported externalizing problems, more parent-reported internalizing problems, and higher CBCL total scores at age 17 but not at age 14 after controlling for parental variables. In contrast, three studies reported lower CBCL total scores in ART children [79, 137, 138], though the effects were limited to specific subsamples or parent reports [79, 137].

The Strengths and Difficulties Questionnaire (SDQ) [139, 140] was administered in six studies, with varying results. Golombok’s research group [59, 82] reported comparable scores in approx. 12-year-old ART and NC children according to mother and teacher reports. Eisemann et al. [141] also found comparable SDQ scores among 14–18-year-old ICSI and NC children based on self-report. In the study by Shelton et al. [142], ART and NC mothers rated their 5–9-year-old children similarly, while ART fathers reported lower conduct problems and prosocial behavior than did NC fathers. Barbuscia et al. [143] found lower SDQ total scores in ART (and other infertility treatment) children initially, but higher scores after controlling for parental variables. These differences decreased with age and were no longer statistically significant by age 14. Carson et al. [144] also revealed higher SDQ scores in ART children at ages five and seven; this association increased after adjusting for confounders and was stronger at age five than at age seven [144].

Studies assessing behavioral problems using the Eyberg Child Behavior Inventory (ECBI) [145] found either no significant differences [19] or fewer behavioral problems in ART children than in NC children [146]. Scores on the Vineland Adaptive Behavior Scales [147, 148] revealed similar daily living skills and similar or higher socialization skills in ART children than in NC children [74, 109]. Golombok’s research group assessed psychosocial problems using the Rutter scales [149] and found no differences between 4–8-year-old ART and NC children [58, 70, 81].

Parents’ reports of their child’s psychosocial development assessed using other measures mainly suggested no difference between ART and NC children [89, 95, 101, 113, 114]. However, Cederblad et al. [115] reported less disturbed behavior in IVF than in NC children, and van Balen [76] found that IVF children were characterized as more social and less obstinate by their mothers but not by their fathers. Only Golombok et al. [150] reported a higher incidence of behavioral and emotional problems in IVF than in NC children. Social relationships with adults and peers appeared to be similar in NC and ART children aged 2–18 years [59, 82, 84, 107, 108].

The majority of teachers’ reports revealed similar psychosocial development in ART and NC children [19, 128]; however, more socioemotional adjustment problems [95] and better compliance to set limits were found in ART than in NC children [19]. The meta-analyses showed comparable or better psychosocial development of ART children compared to NC children (CBCL: k = 9, *p* = 0.089; YSR: k = 3, *p* = 0.656; TRF: k = 3, *p* = 0.513; SDQ: k = 4, *p* =  < 0.001***; Vineland – Socialization: k = 2, *p* = 0.022*; ECBI: k = 2, *p* = 0.002**).

Regarding *mental health*, sixteen studies reported a comparable risk of mental disorders [58, 82, 84, 138, 151] such as obsessive–compulsive disorder (OCD), attention deficit hyperactivity disorder (ADHD) [115, 125, 127], and autism spectrum disorder (ASD) [109, 117, 127, 142, 152–156] in NC and ART children after controlling for parental characteristics. Unadjusted analyses revealed a higher risk of ASD [152, 153] and OCD [151], a lower risk of ADHD [125], and a lower median diagnosis age and symptom severity of ASD in ART children [154].

Ten studies found a slightly higher risk of certain mental health problems [157] in ART children, including tic disorders [116], medically treated ADHD [158], ASD [80, 159, 160], feeding problems [61], aggression [95], anxiety, and depression [95, 136, 161]. In the studies that examined several mental disorders within one study, there were no significant differences in other prevalences [61, 80, 116, 136, 161]. Rissanen et al. [157] reported that ART children had fewer mental disorder diagnoses than NC children, but received their diagnoses earlier. However, after adjusting for confounding factors, ART children had a slightly increased likelihood of any psychiatric diagnosis [157]. In contrast, Eisemann et al. [141] and Hammarberg et al. [162] found higher self-reported quality of life in ART adolescents and adults compared to NC offspring.

## Discussion

In this systematic review and meta-analysis, we investigated possible psychological outcomes of successful ART for the family based on 89 peer-reviewed studies. The literature is heterogenous with regard to outcome measures, populations, timing, and quality. Overall, however, it suggests that ART and NC families may exhibit few differences in family functioning beyond the first postpartum year (Table 4). This aligns with prior reviews (Supplement 1) and studies on ART and NC families during the first postpartum year, which likewise reported few differences between the groups [e.g. 41, 42, 163].

**Fig. 3:**
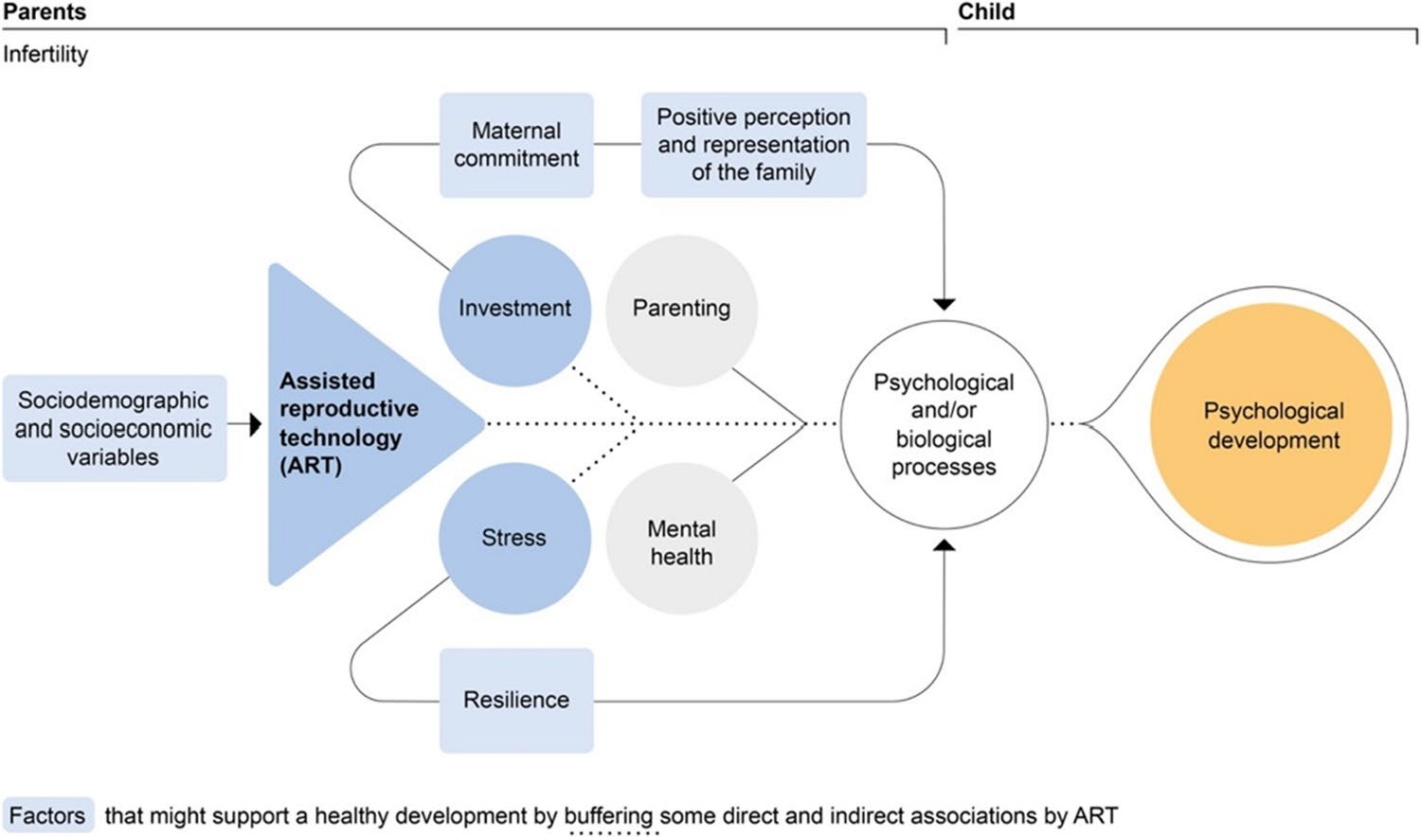
Potential reasons for healthy development of ART families

The healthy family functioning indicates that, contrary to Fig. 1 in the introduction, ART, associated stress, and investment might have little impact on the family. For example, despite birth-related risks of lower child development associated with ART, for instance due to preterm birth [38], ART and NC children show comparable development. Consequently, the effects of infertility, ART, stress, and investment on parental mental health, parenting, and child development, as hypothesized in the introduction, seem to be mitigated. The reviewed studies assumed various mechanisms, which we summarized into four potential buffering factors: sociodemographic and socioeconomic variables, resilience, maternal commitment, and positive perception and representation of the family. Figure 3 presents these factors (light blue boxes) and the mitigated associations (dashed lines).

Couples who opted for ART differ from NC couples in terms of sociodemographic and socioeconomic variables that might buffer the effects of infertility, ART, and stress (Fig. 3). ART parents are on average older, better educated, have higher incomes, higher SES, and are more likely to be primiparous, in (longer) relationships, and married [e.g. 105, 106, 119, 121, 124, 127, 138]. These factors may provide protection against maternal mood disorders and support maternal mental health after ART [42]. High maternal education and income may also promote positive parenting and child outcomes [66, 164]. Notably, in some studies, controlling for parental variables altered the effect sizes or even reversed findings of the comparison between ART and NC families, especially for children’s development. For instance, ART children initially showed better cognitive and psychosocial development, but this diminished or reversed after adjustment [e.g. 83, 105, 125, 143]. Our descriptive analysis confirmed that studies reporting lower development in ART children tended to control for more key covariates (e.g. parental age) than those reporting no differences or better development. However, the effects of parental variables are complex (e.g. maternal age [165, 166]).

Couples undergoing ART often experience stress [e.g. 8], which might strengthen their resilience [167]. In ART mothers, this is supported by findings that a higher number of ART cycles is associated with less parenting stress, better marital adjustment, and better mental health [66, 168, 169]. Additionally, higher resilience seems to increase the likelihood of starting and completing treatment [66, 170, 171], while psychological burden appears to reduce this likelihood [172–175]. If ART parents indeed exhibit high resilience, as hypothesized but not verified by various authors [66, 170, 171], this could support healthy family development (Fig. 3). For example, stress during treatment might enhance resilience to parenting stress [79].

Infertility and ART demand a high financial, time, and emotional investment for couples to become parents [9] (Fig. 3), which likely explains high maternal commitment in ART mothers [176]. Examples include frequent reading to the children [83] and providing them with preschool enrichment programs [19]. This commitment may buffer the effects of infertility, ART, and stress on mothers’ mental health, parenting, and child development (Fig. 3). Its impact on family life was discussed critically in the analyzed studies but usually interpreted rather positively, reflecting a child-centered focus in family life [e.g. 19, 59]. It may contribute to maternal happiness after the long-awaited birth [10, 41], leading to good mental health [177] and low parenting stress [66, 74]. Furthermore, it might promote good mother–child attachment [18, 178], which Bergman et al. [179, 180] found to mitigate the effects of prenatal stress on child development. Additionally, some authors speculated that highly committed ART mothers may provide their children with intellectually stimulating environments [74, 102, 119, 120], interact with them frequently [83, 106, 107], and monitor their health carefully [157, 176], promoting children’s development [97, 118]. High levels of interaction might explain ART children’s slightly better language development [107], and frequent healthcare utilization might explain the slightly higher likelihood of a mental disorder diagnosis [157]. Similar mechanisms in fathers cannot be deduced based on the few previous studies on paternal commitment. The generally smaller differences between ART and NC fathers compared to ART and NC mothers may be due to lower emotional involvement in infertility and ART among fathers [76, 181, 182].

High maternal commitment might reduce the degree to which ART mothers recognize and admit family difficulties [e.g. 80], potentially masking negative ART effects (Fig. 3). In line with the theory of cognitive dissonance, high commitment might lead to overvaluation or idealization of motherhood and children [42, 107, 183]. Boz et al. [184] reported that ART mothers struggle to distance themselves from the idea of being infertile, potentially leading to overcompensation in motherhood and a greater tolerance of behavior that other mothers find challenging [e.g. 146]. Consistent with this, we observed discrepancies between informants: Father and child reports showed no parenting differences, while mothers’ reports favored ART mothers (Table 4). Similarly, according to parents’ reports, ART children have rather better psychosocial development than NC children, while register data suggest that ART children tend to have poorer mental health. Evidence also indicates that in studies, ART parents are less open and less likely to express negative feelings compared to NC parents, potentially masking ART’s negative effects [185].

To summarize, as the studies analyzed are observational, differences and similarities between ART and NC families cannot be definitively attributed to the mode of conception. As illustrated in Fig. 3, factors possibly associated with ART – such as treatment-related stress or sociodemographic variables – may influence family functioning. Consequently, the findings of this review should be interpreted within the broader context of these associated factors.

Legal, societal, and medical developments in ART underline the importance of ongoing research, yet findings on its psychological outcomes remain partly contradictory, and recent research is lacking. The average quality rating of 47% suggests mediocre study quality, reflecting three main issues that likely contributed to divergent results: First, inconsistent control for parental (e.g. parental SES) and birth-specific variables led to varying results [e.g. 83, 106]. Future research should consider these variables in order to identify protective or risk factors for family functioning. This could also include variables that have so far received little attention, such as the parents’ current desire to have children, attempts to conceive another child, or the age of siblings. Consistent reporting of these variables would allow future meta-analyses to systematically assess their influence. Second, most studies did not consider factors that potentially influence stress (e.g. infertility duration, treatment type, number of cycles, biological relationship) [186], despite conflicting evidence on their effects (e.g. treatment cycles [66, 187–189]). Third, most studies did not clarify whether differences stem from ART, infertility, or both, for example by omitting NC parents’ infertility history. Another factor that may have contributed to the differing results is the timing of analysis, as shown by our descriptive analysis: On average, studies reporting positive parenting outcomes in ART families were conducted earlier (mean 4 years postpartum) than those reporting negative outcomes (mean 5 years) or no differences (mean 7 years). Similarly, studies reporting better cognitive and psychosocial development in ART children or no differences were conducted earlier (mean 7–8 years) than those reporting poorer development (mean 11 years). The high heterogeneity in six of the 18 meta-analyses likely reflects these issues. Interestingly, our descriptive analysis revealed that study quality may influence the results, as for parental outcomes, average study quality was highest in the studies without significant differences, whereas for child outcomes, average study quality was highest in studies that indicated lower development of ART children. Finally, it should be noted that many of the included studies relied on self-reported data regarding the use of ART treatment, as registry data and hospital records are not available or accessible in all cases. Although self-reporting is necessary for such studies and seems to show good agreement with registry data, it has limitations and may be prone to misclassification of some individuals [195–197].

Taken together, our results may reassure couples considering ART, as we found no major differences in family functioning. The differences found are small, and probably of little practical relevance, which should be examined in high-quality original studies. However, as the hypothesized high maternal commitment might influence healthcare service use, ART parents should be informed about healthy child development. Reducing stigmatization is crucial to ensure that ART parents report difficulties, both in research and in everyday life (e.g. problems in school), and thus receive appropriate support.

This review is the first to systematically and quantitatively examine the possible psychological outcomes of successful ART for parents’ mental health, parenting, and children’s development together. We highlighted potential associations of ART with different aspects of family life, hypothesized reasons for comparable family development, and may have contributed to the understanding of ART’s influence on the family. However, eight of 18 meta-analyses did not meet the robustness criteria and should be interpreted with caution. Lack of data prevented the inclusion of all relevant studies, leaving the robustness of some effects, particularly regarding fathers’ STAI and mothers’ PSI scores, uncertain. Additionally, high heterogeneity in some meta-analyses (e.g. mothers’ mental health) reflects contradictory findings, complicating interpretation. Furthermore, when interpreting the results, it should be noted that random-effects meta-analyses were conducted based on theoretical considerations; however, in analyses including only a few studies, this approach has limitations, as the between-study variance may not be estimated accurately [54]. Finally, given the range of outcomes examined, some from the same studies, multiplicity and the possibility of chance findings should be considered when interpreting statistically significant results. In view of these limitations, the results of the meta-analyses should not be interpreted in isolation, but rather in combination with the results of the systematic review, with a stronger emphasis on the latter, as presented in Table 4.

## Conclusion

Achieving parenthood through ART is stressful and requires a high level of investment [8]. More than a year after birth, ART families appear to have adapted well to family life, potentially due to factors that buffer the effects of infertility, ART, and stress (Fig. 3). ART parents may exhibit protective sociodemographic and socioeconomic characteristics, high resilience, and strong maternal commitment. The latter may explain the positive findings that only emerged in mothers’ reports, as ART mothers may perceive or portray parenthood and their children especially positively. As the children tend to develop healthily, this commitment appears to have no negative impact on them. Discrepancies between different informants’ reports highlight the importance of including objective measures, such as stress biomarkers. To inform the development of interventions, future research should address methodological limitations evident in the existing literature and identify parental characteristics and birth risks that amplify or buffer differences between ART and NC families.

## Supplementary Information

Below is the link to the electronic supplementary material.

Supplementary file1 (DOCX 65 KB)

Supplementary file2 (DOCX 45 KB)

Supplementary file3 (DOCX 133 KB)

## Data Availability

Additional data underlying this article are available from the corresponding author on reasonable request.
