# Supplementary material for: Successful assisted reproduction treatment and its psychological outcomes for parents and children: a systematic review and meta-analysis
Source: J Assist Reprod Genet. 2025 Jul 9;42(9):2817–36. doi: 10.1007/s10815-025-03572-9 (PMC12559479; doi:10.1007/s10815-025-03572-9)
Supplement: Supplementary file 1 — Supplementary file1 (DOCX 65 KB) [file 10815_2025_3572_MOESM1_ESM.docx]

# Supplement 1

## Tables showing the characteristics, outcomes, and main findings of previous reviews regarding psychological family functioning after successful assisted reproduction

**Supplementary Table S1** Main findings of previous reviews regarding psychological family functioning after successful assisted reproduction

|  | **Main findings regarding psychological family functioning after successful assisted reproduction** |
| --- | --- |
| McMahon et al., 1995 | Comparable parenting and cognitive and psychosocial child development in IVF and NC families; Limited studies and significant methodological shortcomings highlight need for future high-quality research |
| Van Balen, 1998 | Comparable cognitive, psychomotor, and psychosocial child development and indication of better mother-child relationship in IVF than NC families |
| Hahn, 2001 | Comparable cognitive and psychosocial child development and indication of less parenting stress and better parent-child relationship in ART than NC families; Studies show methodological shortcomings |
| Colpin, 2002 | Comparable psychosocial child development and comparable or better parent-child relationship in ART than NC families; Studies show methodological shortcomings |
| Greenfeld & Klock, 2005 | Comparable parenting and psychosocial development in ART and NC children |
| Hammarberg et al., 2008 | Body of evidence regarding family functioning after ART is best described as emergent; Selection of study and comparison group is crucial |
| Izat & Goldbeck, 2008 | Comparable cognitive, psychomotor, and psychosocial development in ART and NC children; Data are preliminary |
| Middelburg et al., 2008 | Comparable cognitive, psychomotor, and psychosocial development in ART and NC children; Further high-quality research needed |
| Wagenaar et al., 2008a | Comparable parent-child relationship, parenting behavior and cognitive, psychomotor, and psychosocial child development, and indication of more parenting difficulties in the first year after birth and more parental emotional involvement in later years in ART than NC families; More research in adolescence needed |
| Hvidtjørn et al., 2009 | Inconsistent findings regarding cognitive, psychomotor, and psychosocial development and risk of ASD in ART and NC children; Studies show methodological shortcomings; Further studies on long-term outcomes in ART children needed |
| McGrath et al., 2010 | Inconsistent findings regarding parenting after ART; ART-associated risk factors (e.g. maternal age) should be further investigated |
| Ross et al., 2011 | Comparable risk of postpartum depression among ART and NC mothers; Research considering covariates like multiple births needed |
| Wilson et al., 2011 | Comparable parent-child relationship and cognitive, psychomotor, and psychosocial child development in ART and NC families; Further high-quality research needed, especially in young adults |
| Bay et al., 2013 | Comparable cognitive, psychomotor, and psychosocial development in ART and NC children; Further high-quality research needed (e.g. mental health) |
| Conti et al., 2013 | Comparable risk of ASD in ART and NC children; Further high-quality research needed |
| Wang et al., 2014 | Comparable warmth, rejection, and respect for autonomy in parenting but more controlling parenting behaviors in IVF than NC mothers; Future research should consider personal variables (e.g. prematurity) |
| Ilioi & Golombok, 2015 | Comparable parent-child relationship and psychosocial development in ART and NC children; Further follow-up studies needed |
| Turkgeldi et al., 2016 | Comparable cognitive, psychomotor, and psychosocial development in ART and NC children; Further follow-up studies needed |
| Rumbold et al., 2017 | Comparable cognitive development of IVF and NC children but inconsistent findings regarding the comparison of ICSI and NC children; Further high-quality research needed |
| Pereira et al., 2017 | Comparable cognitive, psychomotor, and psychosocial development in ICSI and NC children; The association between ICSI and ASD should be further investigated; Future research should consider covariates (e.g. maternal age, multiple births, SES) |
| Allan et al., 2019 | Differences in the transition from pregnancy to parenthood regarding social support, relationships, and emotional well-being in ART and NC families; Future research should pay more attention to fathers |
| Bergh & Wennerholm, 2020 | Comparable cognitive, psychomotor, and psychosocial child development in ART and NC families when restricted to singletons; Further high-quality follow-up studies needed; Selection of study and comparison group is crucial |
| Capuzzi et al., 2020 | Comparable risk of perinatal affective symptoms in IVF and NC mothers; Further high-quality research considering covariates and stress biomarkers needed; Meta-analysis recommended |
| Djuwantono et al., 2020 | Comparable risk of intellectual disability, ASD, and behavioral problems in ART and NC children; Further high-quality follow-up studies considering covariates such as preterm birth needed |
| Andreadou et al., 2021 | Higher risk of ASD in ART than NC children, but not when comparison restricted to singletons; Further high-quality prospective studies considering covariates (e.g. higher maternal age) needed |
| Gullo et al., 2022 | Comparable school performance and risk of neurodevelopmental diseases and in ART and NC children; Further high-quality research considering covariates such as pre-term birth and multiple pregnancies needed |
| Jenabi et al., 2022 | Comparable risk of ASD in ART and NC children; Further high-quality research needed |
| Carneiro et al., 2022 | Psychosocial development of ART children was within the normal range of normative data |
| Quintigliano et al., 2022 | Comparable parent-child relationship and cognitive, psychomotor, and psychosocial development in ART and NC children; Further research investigating child development in diverse ART families needed |
| Djuwantono et al., 2023 | Differences in cognitive, psychomotor, and psychosocial development between ART and NC children, depending on children’s age; Covariates such as prematurity, SES, and parenting style may influence results |
| Graham et al., 2023 | Comparable cognitive development and risk of ASD in ART and NC children after controlling for multiple gestations and prematurity; Further long-term high-quality studies needed |
| Pinborg et al., 2023 | Comparable cognitive development and risk of ASD and ADHD in ART and NC children when controlling for parental factors and multiple births |
| Ono et al., 2024 | Comparable neurodevelopment such as school performance and risk of ASD and ADHD in ART and NC children; Variables like parents’ genetic makeup may play a significant role in child development after ART |
| Zeng et al., 2024 | Conflicting data regarding ASD, intellectual disability, ADHD in ART and NC children due to covariates (e.g. multiple birth); Further high-quality research considering covariates needed |
| To the best of our knowledge, the table includes all reviews that compared families after successful assisted reproduction with NC families regarding psychological functioning. The main outcomes of the reviews had to include at least one psychological aspect. ADHD, attention deficit hyperactivity disorder; ASD, autism spectrum disorder; NC, natural conception; SES, socioeconomic status. | |

**Supplementary Table S2** Characteristics and outcomes of previous reviews regarding psychological family functioning after successful assisted reproduction

|  | **Review characteristics** | | | | | **Outcomes of interest** | | | | | | | | | |  |
| --- | --- | --- | --- | --- | --- | --- | --- | --- | --- | --- | --- | --- | --- | --- | --- | --- |
|  | >5 yrs old | SR | MA | QR | Model ^a^ | | Mental health mother | Mental health father | Parents’ relationship | Parenting stress | Parent-child relationship | Parenting behavior | Cognitive development | Psychomotor development | Psychosocial development and mental health | |
| McMahon et al., 1995 |  |  |  |  |  | | X |  | X | X | X | X | X |  | X | |
| Van Balen, 1998 |  |  |  |  |  | |  |  |  |  | X |  | X | X | X | |
| Hahn, 2001 |  |  |  |  |  | |  |  | X | X | X | X | X |  | X | |
| Colpin, 2002 |  | X |  |  |  | | X | X | X | X | X | X |  |  | X | |
| Greenfeld & Klock, 2005 |  |  |  |  |  | | X | X | X | X | X | X |  |  | X | |
| Hammarberg et al., 2008 |  | X |  |  |  | | X | X | X | X | X | X |  |  |  | |
| Izat & Goldbeck, 2008 |  | X |  |  |  | |  |  |  |  |  |  | X | X | X | |
| Middelburg et al., 2008 |  | X |  | X |  | |  |  |  |  |  |  | X | X | X | |
| Wagenaar et al., 2008a |  |  |  |  |  | |  |  |  | X | X | X | X | X | X | |
| Hvidtjørn et al., 2009 |  | X |  |  |  | |  |  |  |  |  |  | X | X | X | |
| McGrath et al., 2010 |  |  |  |  |  | | X | X | X | X | X | X |  |  |  | |
| Ross et al., 2011 |  | X |  | X |  | | X |  |  |  |  |  |  |  |  | |
| Wilson et al., 2011 |  |  |  |  |  | |  |  |  |  | X |  | X | X | X | |
| Bay et al., 2013 |  | X |  | X |  | |  |  |  |  |  |  | X | X | X | |
| Conti et al., 2013 |  | X |  | X |  | |  |  |  |  |  |  |  |  | X | |
| Wang et al., 2014 |  | X | X | X |  | |  |  |  |  |  | X |  |  |  | |
| Ilioi & Golombok, 2015 |  | X |  |  |  | |  |  |  |  | X |  |  |  | X | |
| Turkgeldi et al., 2016 |  |  |  |  |  | |  |  |  |  |  |  | X | X | X | |
| Rumbold et al., 2017 |  | X |  | X |  | |  |  |  |  |  |  | X |  |  | |
| Pereira et al., 2017 |  |  |  |  |  | |  |  |  |  |  |  | X | X | X | |
| Allan et al., 2019 |  | X |  | X |  | | X | X | X | X | X | X |  |  | X | |
| Bergh & Wennerholm, 2020 | X |  |  |  |  | |  |  |  |  |  |  | X | X | X | |
| Capuzzi et al., 2020 | X | X |  | X |  | | X |  |  |  |  |  |  |  |  | |
| Djuwantono et al., 2020 | X | X | X | X |  | |  |  |  |  |  |  | X |  | X | |
| Andreadou et al., 2021 | X | X | X | X |  | |  |  |  |  |  |  |  |  | X | |
| Gullo et al., 2022 | X |  |  |  |  | |  |  |  |  |  |  | X | X | X | |
| Jenabi et al., 2022 | X | X | X | X |  | |  |  |  |  |  |  |  |  | X | |
| Carneiro et al., 2022 | X | X |  | X |  | |  |  |  |  |  |  |  |  | X | |
| Quintigliano et al., 2022 | X |  |  |  |  | |  |  |  |  | X |  | X | X | X | |
| Djuwantono et al., 2023 | X | X | X | X |  | |  |  |  |  |  |  | X | X | X | |
| Graham et al., 2023 | X |  |  |  | X | |  |  |  |  |  |  | X |  | X | |
| Pinborg et al., 2023 | X |  |  |  |  | |  |  |  |  |  |  | X |  | X | |
| Ono et al., 2024 | X |  |  |  |  | |  |  |  |  |  |  | X | X | X | |
| Zeng et al., 2024 | X |  |  |  | X | |  |  |  |  |  |  | X | X | X | |
| ^a^ Associations are illustrated in a model  To the best of our knowledge, the table includes all reviews that compared families after successful assisted reproduction with NC families regarding psychological functioning. The main outcomes of the reviews had to include at least one psychological aspect. MA, meta-analysis; SR, systematic review; QR, quality rating; yrs, years. | | | | | | | | | | | | | | | | |

**References**

Allan HT, van den Akker O, Culley L, Mounce G, Odelius A, Symon A. An integrative literature review of psychosocial factors in the transition to parenthood following non-donor-assisted reproduction compared with spontaneously conceiving couples. Hum Fertil. 2019;1–18. https://doi.org/10.1080/14647273.2019.1640901.

Andreadou MT, Katsaras GN, Talimtzi P, Doxani C, Zintzaras E, Stefanidis I. Association of assisted reproductive technology with autism spectrum disorder in the offspring: an updated systematic review and meta-analysis. Eur J Pediatr. 2021;180:2741–55. https://doi.org/10.1007/s00431-021-04187-9.

Bay B, Mortensen EL, Kesmodel US. Assisted reproduction and child neurodevelopmental outcomes: a systematic review. Fertil Steril. 2013;100:844–53. https://doi.org/10.1016/j.fertnstert.2013.05.034.

Bergh C, Wennerholm UB. Long-term health of children conceived after assisted reproductive technology. Ups J Med Sci. 2020;125:152–7. https://doi.org/10.1080/03009734.2020.1729904.

Capuzzi E, Caldiroli A, Ciscato V, Zanvit FG, Bollati V, Barkin JL, Clerici M, Buoli M. Is in vitro fertilization (IVF) associated with perinatal affective disorders? J Affect Disord. 2020;277:271–8. https://doi.org/10.1016/j.jad.2020.08.006.

Carneiro FAT, Leong V, Nóbrega S, Salinas-Quiroz F, Costa PA, Leal I. Are the children alright? A systematic review of psychological adjustment of children conceived by assisted reproductive technologies. Eur Child Adolesc Psychiatry. Advance Access published Dec 29, 2022. https://doi.org/10.1007/s00787-022-02129-w.

Colpin H. Parenting and psychosocial development of IVF children: Review of the research literature. Dev Rev. 2002;22:644–73.

Conti E, Mazzotti S, Calderoni S, Saviozzi I, Guzzetta A. Are children born after assisted reproductive technology at increased risk of autism spectrum disorders? A systematic review. Hum Reprod. 2013;28:3316–27. https://doi.org/10.1093/humrep/det380.

Djuwantono T, Aviani JK, Permadi W, Achmad TH, Halim D. Risk of neurodevelopmental disorders in children born from different ART treatments: a systematic review and meta-analysis. J Neurodev Disord. 2020;12:33. https://doi.org/10.1186/s11689-020-09347-w.

Djuwantono T, Aviani JK, Permadi W, Halim D, Achmad TH, Dhamayanti M. Intelligence, motoric and psychological outcomes in children from different ART treatments: a systematic review and meta-analysis. J Neurodev Disord. 2023;15:26. https://doi.org/10.1186/s11689-023-09490-0.

Graham ME, Jelin A, Hoon AH Jr, Wilms Floet AM, Levey E, Graham EM. Assisted reproductive technology: Short- and long-term outcomes. Dev Med Child Neurol. 2023;65:38–49. https://doi.org/10.1111/dmcn.15332.

Greenfeld DA, Klock SC. Children of assisted reproductive technology: medical and psychological issues. Psychol J Hellenic Psychol Soc. 2005;12:5–14.

Gullo G, Scaglione M, Cucinella G, Perino A, Chiantera V, D'Anna R, Laganà AS, Buzzaccarini G. Impact of assisted reproduction techniques on the neuro-psycho-motor outcome of newborns: a critical appraisal. J Obstet Gynaecol. 2022;42:2583–7. https://doi.org/10.1080/01443615.2022.2109953.

Hahn CS. Review: psychosocial well-being of parents and their children born after assisted reproduction. J Pediatr Psychol. 2001;26:525–38. https://doi.org/10.1093/jpepsy/26.8.525.

Hammarberg K, Fisher JR, Wynter KH. Psychological and social aspects of pregnancy, childbirth and early parenting after assisted conception: a systematic review. Hum Reprod Update. 2008;14:395–414. https://doi.org/10.1093/humupd/dmn030.

Hvidtjørn D, Schieve L, Schendel D, Jacobsson B, Svaerke C, Thorsen P. Cerebral palsy, autism spectrum disorders, and developmental delay in children born after assisted conception: a systematic review and meta-analysis. Arch Pediatr Adolesc Med. 2009;163:72–83. https://doi.org/10.1001/archpediatrics.2008.507.

Ilioi EC, Golombok S. Psychological adjustment in adolescents conceived by assisted reproduction techniques: a systematic review. Hum Reprod Update. 2015;21:84–96. https://doi.org/10.1093/humupd/dmu051.

Izat Y, Goldbeck L. Die Entwicklung von Kindern aus assistierten Befruchtungen: Eine Übersicht der Studienlage. Praxis der Kinderpsychologie und Kinderpsychiatrie. 2008;57:264–81.

Jenabi E, Bashirian S, Khazaei S, Farhadi Nasab A, Maleki A. The Association between Assisted Reproductive Technology and the Risk of Autism Spectrum Disorders among Offspring: A Meta-analysis. Curr Pediatr Rev. 2022;19:83–9. https://doi.org/10.2174/1573396318666220410231435.

McGrath JM, Samra HA, Zukowsky K, Baker B. Parenting after infertility: issues for families and infants. MCN Am J Matern Child Nurs. 2010;35:156–64. https://doi.org/10.1097/NMC.0b013e3181d7657d.

McMahon CA, Ungerer JA, Beaurepaire J, Tennant C, Saunders D. Psychosocial outcomes for parents and children after in vitro fertilization: a review. J Reprod Infant Psychol. 1995;13:1–16. https://doi.org/10.1080/02646839508403227.

Middelburg KJ, Heineman MJ, Bos AF, Hadders-Algra M. Neuromotor, cognitive, language and behavioural outcome in children born following IVF or ICSI-a systematic review. Hum Reprod Update. 2008;14:219–131. https://doi.org/10.1093/humupd/dmn005.

Ono M, Kuji N, Ueno K, Kojima J, Nishi H. The Long-Term Outcome of Children Conceived Through Assisted Reproductive Technology. Reprod Sci. 2024;31:583–90. https://doi.org/10.1007/s43032-023-01339-0.

Pinborg A, Wennerholm UB, Bergh C. Long-term outcomes for children conceived by assisted reproductive technology. Fertil Steril. 2023;120:449–56. https://doi.org/10.1016/j.fertnstert.2023.04.022.

Pereira N, O'Neill C, Lu V, Rosenwaks Z, Palermo GD. The safety of intracytoplasmic sperm injection and long-term outcomes. Reproduction. 2017;154:F61–F70. https://doi.org/10.1530/REP-17-0344.

Quintigliano M, Carone N, Speranza AM, Tanzilli A, Baiocco R, Barone L, Pastorelli C, Lingiardi V. Adolescent Development and the Parent-Adolescent Relationship in Diverse Family Forms Created by Assisted Reproduction. Int J Environ Res Public Health. 2022;19:16758. https://doi.org/10.3390/ijerph192416758.

Ross LE, McQueen K, Vigod S, Dennis CL. Risk for postpartum depression associated with assisted reproductive technologies and multiple births: a systematic review. Hum Reprod Update. 2011;17:96–106. https://doi.org/10.1093/humupd/dmq025.

Rumbold AR, Moore VM, Whitrow MJ, Oswald TK, Moran LJ, Fernandez RC, Barnhart KT, Davies MJ. The impact of specific fertility treatments on cognitive development in childhood and adolescence: a systematic review. Hum Reprod. 2017;32:1489–507. https://doi.org/10.1093/humrep/dex085.

Turkgeldi E, Yagmur H, Seyhan A, Urman B, Ata B. Short and long term outcomes of children conceived with assisted reproductive technology. Eur J Obstet Gynecol Reprod Biol. 2016;207:129–36. https://doi.org/10.1016/j.ejogrb.2016.10.010.

Van Balen F. Development of IVF children. Developmental Review. 1998;18:30–46. https://doi.org/10.1006/drev.1997.0446.

Wagenaar K, Huisman J, Cohen-Kettenis PT, Delemarre-van de Waal HA. An overview of studies on early development, cognition, and psychosocial well-being in children born after in vitro fertilization. J Dev Behav Pediatr. 2008a;29:219–30. https://doi.org/10.1097/DBP.0b013e318173a575.

Wang YM, Shu BC, Fetzer S, Chang YJ. Parenting style of women who conceived using in vitro fertilization: a meta-analysis. J Nurs Res. 2014;22:69–80. https://doi.org/10.1097/JNR.0000000000000025.

Wilson CL, Fisher JR, Hammarberg K, Amor DJ, Halliday JL. Looking downstream: a review of the literature on physical and psychosocial health outcomes in adolescents and young adults who were conceived by ART. Hum Reprod. 2011;26:1209–19. https://doi.org/10.1093/humrep/der041.

Zeng Z, Wang Z, Yu P, Wang Y, Pei Y, Dai Y, Liu Y, Yang Y. The Association between Assisted Reproductive Technologies and Neurodevelopmental Disorders in Offspring: An Overview of Current Evidence. J Integr Neurosci. 2024;23:15. https://doi.org/10.31083/j.jin2301015.
