# Supplementary material for: Successful assisted reproduction treatment and its psychological outcomes for parents and children: a systematic review and meta-analysis
Source: J Assist Reprod Genet. 2025 Jul 9;42(9):2817–36. doi: 10.1007/s10815-025-03572-9 (PMC12559479; doi:10.1007/s10815-025-03572-9)
Supplement: Supplementary file 2 — Supplementary file2 (DOCX 45 KB) [file 10815_2025_3572_MOESM2_ESM.docx]

# Supplement 2

## Quality assessment scale

Based on Tak et al. (2011), Fischer and Cleare (2017), and Amiel Castro et al. (2021) ^[[1]](#footnote-1)^

*16 resp. 14 points equal 100%.*

Appropriate selection and description of participants

1. What eligibility criteria were used?

No major congenital abnormalities or chronic illness of the children, no very premature (gestational age ≤32 weeks) or very low birth weight (birth weight ≤1500 g) children, 2 used (2)

No major congenital abnormalities or chronic illness of the children, no very premature (gestational age ≤32 weeks) or very low birth weight (birth weight ≤1500 g) children, 1 used (1)

No major congenital abnormalities or chronic illness of the children, no very premature (gestational age ≤32 weeks) or very low birth weight (birth weight ≤1500 g) children, 0 used or not clearly stated (0)

1. How was the NC group recruited?

From the same population as the ART group (e.g. same hospital) (2)

From another population (e.g. single hospital vs. specific school) or not clearly stated (0)

1. Are details given about infertility and infertility treatments of the ART group?

Cause of infertility, duration of infertility, type of infertility treatment, number of treatment cycles, sperm donation, oocyte donation, 4-6 stated (2)

Cause of infertility, duration of infertility, type of infertility treatment, number of treatment cycles, sperm donation, oocyte donation, 2-3 stated (1)

Cause of infertility, duration of infertility, type of infertility treatment, number of treatment cycles, sperm donation, oocyte donation, 0-1 stated (0)

1. Is it stated whether the control group conceived within 12 months and whether they had ever used infertility treatments?

Time to conceive *and* use of infertility treatments stated (2)

Time to conceive *or* use of infertility treatments stated (1)

Neither time to conceive nor use of infertility treatments stated (0)

Appropriate quantification of outcome

1. Has the outcome been reliably assessed?

Validated interview or test by a trained study member (2)

Validated self-report, parents' report, or teacher's report questionnaire (1)

Non-validated questionnaire, non-validated interview, or not clearly stated (0)

1. Is the assessor of outcome blind to group membership?

Yes (2)

No or not clearly stated (0)

Not applicable (/)

1. Are the relevant statistics presented?

Sample size, mean value, and standard deviation available (2)

Sample size and frequency table available (2)

Only sample size available or only mean value and standard deviation available (1)

Only sample size available or only frequency table available (1)

Relevant statistics not clearly stated (0)

Appropriate control for confounders

1. Are the analyses adjusted for potential confounders?

*In the case of matched groups, the variable being an exclusion criterion, or in case of no significant impact on statistical analyses, confounders were considered as adjusted for.*

Child's gender/sex, child’s age, family size / parity, multiple births, parent's age (at least from one parent), socioeconomic status (parental education / parental income at least from one parent), marital status, premature birth / gestational week / birth weight, 6-8 stated (2)

Child's gender/sex, child’s age, family size / parity, multiple births, parent's age (at least from one parent), socioeconomic status (parental education / parental income at least from one parent), marital status, premature birth / gestational week / birth weight, 3-5 stated (1)

Child's gender/sex, child’s age, family size / parity, multiple births, parent's age (at least from one parent), socioeconomic status (parental education / parental income at least from one parent), marital status, premature birth / gestational week / birth weight, 0-2 stated (0)

1. Amiel Castro RT, Ehlert U, Fischer S. Variation in genes and hormones of the hypothalamic-pituitary-ovarian axis in female mood disorders - A systematic review and meta-analysis. Front Neuroendocrinol. 2021;62:100929. https://doi.org/10.1016/j.yfrne.2021.100929.

   Fischer S, Cleare AJ. Cortisol as a predictor of psychological therapy response in anxiety disorders-Systematic review and meta-analysis. J Anxiety Disord. 2017;47:60–8. https://doi.org/10.1016/j.janxdis.2017.02.007.

   Tak LM, Cleare AJ, Ormel J, Manoharan A, Kok IC, Wessely S, Rosmalen JG. Meta-analysis and meta-regression of hypothalamic-pituitary-adrenal axis activity in functional somatic disorders. Biol Psychol. 2011;87:183–94. https://doi.org/10.1016/j.biopsycho.2011.02.002. [↑](#footnote-ref-1)
