# Supplementary material for: Successful assisted reproduction treatment and its psychological outcomes for parents and children: a systematic review and meta-analysis
Source: J Assist Reprod Genet. 2025 Jul 9;42(9):2817–36. doi: 10.1007/s10815-025-03572-9 (PMC12559479; doi:10.1007/s10815-025-03572-9)
Supplement: Supplementary file 3 — Supplementary file3 (DOCX 133 KB) [file 10815_2025_3572_MOESM3_ESM.docx]

# Supplement 3

## Tables with the analyzed studies investigating the possible impact of successful assisted reproduction on parents and child.

**Supplementary Table S3** Mental health of the mother

| **Study** | **Country** | **Study groups** | **Time point** | **Outcome measures** | **Main results** | **Quality rating** | **Data included in MA** |
| --- | --- | --- | --- | --- | --- | --- | --- |
| Barnes et al., 2004 | Belgium, Denmark, Greece, Sweden, UK | 228 ICSI, 214 IVF, 209 NC mothers | 4.5-5.5 yrs pp | GHQ-28 | NS | 43% | ^a^ |
| Colpin et al., 1995 | Belgium | 31 IVF, 31 NC mothers | 2-2.5 yrs pp | ZDS; STAI | NS | 64% | ^a^; STAI |
| Golombok et al., 1995 | UK | 38 IVF, 39 NC mothers  N depends on the variable | 4-8 yrs pp | Interview (psychiatric treatment); BDI; STAI-Trait | Lower BDI and STAI-Trait scores in IVF than NC mothers | 43% | ^a^; BDI; STAI |
| Golombok et al., 2002 | UK, Spain, Italy, Netherlands | 102 IVF, 102 NC mothers | 11-12 yrs pp | BDI; STAI | NS | 29% | ^c^ |
| Kiesswetter et al., 2023 | Italy | 75 IVF, 70 NC mothers  75 IVF, 70 NC fathers | 1.5, 2 yrs pp | LSQ; PSQ; PSWQ | Lower worry in IVF than NC mothers 2 yrs pp | 14% | ^a^ |
| McMahon et al., 2003 | Australia | 66 IVF, 46 NC mothers | 5 yrs pp | GHQ-28; STAI-State; Scale developed by Rosenberg; LCBS; CECS | More external locus of control in IVF than NC mothers | 50% | ^c^ |
| Owen & Golombok, 2009 | UK | 26 IVF, 63 formerly infertile mothers | 17-19 yrs pp | BDI; STAI | NS | 43% | ^b; c^ |
| Özbaran et al., 2013 | Turkey | 35 ART, 35 NC mothers | 2-13 yrs pp | BDI; STAI | Higher BDI and STAI scores in ART than NC mothers | 36% | BDI; STAI |
| Raoul-Duval et al., 1994 | France | 29 IVF, 26 NC mothers  N depends on time point | 1.5, 3 yrs pp | Interview (depression prevalence) | NS | 64% | ^a^ |
| ^a^ Not enough studies with this outcome measure(s) available to conduct a meta-analysis.  ^b^ Excluded from meta-analyses due to overlapping samples.  ^c^ Excluded from meta-analyses due to lack of data.  All group comparisons not reported in the main results were not significant. BDI, Beck Depression Inventory (Beck & Steer, 1987; Beck et al., 1961; Hisli, 1988); CECS, Courtauld Emotional Control Scale (Watson & Greer, 1983); GHQ-28, General Health Questionnaire (Goldberg & Hillier, 1979); LCBS, Locus of Control of Behaviour Scale (Craig et al., 1984); LSQ, Life Satisfaction Questionnaire (Fahrenberg et al., 2000); MA, meta-analyses; NC, natural conception; NS, not significant; pp, postpartum; PSQ, Perceived Stress Questionnaire (Fliege et al., 2001); PSWQ, Penn State Worry Questionnaire (Meyer et al., 1990); STAI, State-Trait Anxiety Inventory (Öner et al., 1985; Spielberger, 1966; Spielberger, 1983; Spielberger et al., 1970; Spielberger et al., 1983; Van der Ploeg, 1980); yrs, years; ZDS, Zung Depression Scale (Zung, 1965). | | | | | | | |

**Supplementary Table S4** Mental health of the father

| **Study** | **Country** | **Study groups** | **Time point** | **Outcome measures** | **Main results** | **Quality rating** | **Data included in MA** |
| --- | --- | --- | --- | --- | --- | --- | --- |
| Barnes et al., 2004 | Belgium, Denmark, Greece, Sweden, UK | 201 ICSI, 194 IVF, 185 NC fathers | 4.5-5.5 yrs pp | GHQ-28 | NS | 43% | ^a^ |
| Colpin et al., 1995 | Belgium | 30 IVF, 29 NC fathers | 2-2.5 yrs pp | ZDS; STAI | NS | 64% | ^a^; STAI |
| Golombok et al., 1995 | UK | 30 IVF, 25 NC fathers | 4-8 yrs pp | Interview (psychiatric treatment); BDI; STAI-Trait | Lower prevalence of psychiatric treatment and lower STAI scores in IVF than NC fathers | 43% | ^a^; STAI |
| Golombok et al., 1996 | UK, Spain, Italy, Netherlands | 116 IVF, 120 NC fathers | 4-8 yrs pp | BDI; STAI-Trait | NS | 29% | ^c^ |
| Golombok et al., 2002 | UK, Spain, Italy, Netherlands | 102 IVF, 102 NC fathers | 11-12 yrs pp | BDI; STAI | NS | 29% | ^b; c^ |
| Kiesswetter et al., 2023 | Italy | 75 IVF, 70 NC fathers | 1.5, 2 yrs pp | LSQ; PSQ; PSWQ | Lower worry in IVF than NC fathers 2 yrs pp | 14% | ^a^ |
| McMahon et al., 2003 | Australia | 58 IVF, 36 NC fathers | 5 yrs pp | GHQ-28; STAI-State; Scale developed by Rosenberg; LCBS; CECS | NS | 50% | ^c^ |
| Owen & Golombok, 2009 | UK | 26 IVF, 63 formerly infertile fathers | 17-19 yrs pp | BDI; STAI | NS | 43% | ^b; c^ |
| Taubman - Ben-Ari et al., 2017 | Israel | 76 ART, 96 NC fathers | 0.5-1.5 yrs pp | SWLS | NS | 50% | ^a^ |
| Taubman - Ben-Ari et al., 2018 | Israel | 76 ART, 96 NC fathers | 0.5-1.5 yrs pp | PTGI; LOT; PANAS | NS | 50% | ^a^ |
| ^a^ Not enough studies with this outcome measure(s) available to conduct a meta-analysis.  ^b^ Excluded from meta-analyses due to overlapping samples.  ^c^ Excluded from meta-analyses due to lack of data.  All group comparisons not reported in the main results were not significant. BDI, Beck Depression Inventory (Beck & Steer, 1987); CECS, Courtauld Emotional Control Scale (Watson & Greer, 1983); GHQ-28, General Health Questionnaire (Goldberg & Hillier, 1979); LCBS, Locus of Control of Behaviour Scale (Craig et al., 1984); LOT, Life Orientation Test (Scheier & Carver, 1985); LSQ, Life Satisfaction Questionnaire (Fahrenberg et al., 2000); NC, natural conception; NS, not significant; PANAS, Positive and Negative Affect Schedule (Watson et al., 1988); pp, postpartum; PSQ, Perceived Stress Questionnaire (Fliege et al., 2001); PSWQ, Penn State Worry Questionnaire (Meyer et al., 1990); PTGI, Posttraumatic Growth Inventory (Tedeschi & Calhoun, 1996); STAI, State-Trait Anxiety Inventory (Spielberger, 1966; Spielberger, 1983; Spielberger et al., 1970; Van der Ploeg, 1980); SWLS, Satisfaction with Life Scale (Diener et al., 1985); yrs, years; ZDS, Zung Depression Scale (Zung, 1965). | | | | | | | |

**Supplementary Table S5** Parents’ relationship

| **Study** | **Country** | **Study groups** | **Time point** | **Outcome measures** | **Main results** | **Quality rating** | **Data included in MA** |
| --- | --- | --- | --- | --- | --- | --- | --- |
| Barnes et al., 2004 | Belgium, Denmark, Greece, Sweden, UK | Mothers: 208 ICSI, 214 IVF, 209 NC  Fathers: 90 ICSI, 101 IVF, 99 NC | 4.5-5.5 yrs pp | DAS | Mothers & fathers: NS | 43% | ^a^ |
| Colpin et al., 1995 | Belgium | Mothers: 31 IVF, 31 NC  Fathers: 30 IVF, 29 NC | 2-2.5 yrs pp | MMQ | Mothers & fathers: NS | 64% | ^a^ |
| Golombok et al., 1995 | UK | Mothers: 35 IVF, 36 NC  Fathers: 30 IVF, 25 NC | 4-8 yrs pp | GRIMS | Mothers & fathers: NS | 43% | ^a^ |
| Golombok et al., 1996 | UK, Spain, Italy, Netherlands | Mothers: 116 IVF, 120 NC  Fathers: 116 IVF, 120 NC | 4-8 yrs pp | GRIMS | Mothers & fathers: NS | 29% | ^c^ |
| Golombok et al., 2002 | UK, Spain, Italy, Netherlands | Mothers: 102 IVF, 102 NC  Fathers: 102 IVF, 102 NC | 11-12 yrs pp | GRIMS | Mothers & fathers: NS | 29% | ^b; c^ |
| Hahn & DiPietro, 2001 | Taiwan | Mothers: 54 IVF, 58 NC | 3-7 yrs pp | PCI | Mothers: NS | 71% | ^a^ |
| McMahon et al., 2003 | Australia | Mothers: 66 IVF, 46 NC  Fathers: 58 IVF, 36 NC | 5 yrs pp | DAS | Mothers: Mothers with higher levels of IVF treatment reported more positive marital adjustment  Fathers: NS | 50% | ^c^ |
| Owen & Golombok, 2009 | UK | Mothers: 26 IVF, 63 formerly infertile  Fathers: 26 IVF, 63 formerly infertile | 17-19 yrs pp | GRIMS | Mothers & fathers: NS | 43% | ^b; c^ |
| Pottinger & Palmer, 2013 | Jamaica | Mothers: 19 ART, 55 NC  Fathers: 3 ART, 5 NC | ≤7 yrs pp | DAS-7 | Mothers & fathers: NS | 14% | ^c^ |
| Sydsjö et al., 2008 | Sweden | Mothers: 13 IVF (twins), 86 IVF (singletons), 157 NC  Fathers:13 IVF (twins), 79 IVF (singletons), 144 NC | 5 yrs pp | ENRICH | Mothers & fathers: IVF singleton parents had the most stable relationship | 29% | ^c^ |
| Taubman - Ben-Ari et al., 2017 | Israel | Fathers: 76 ART, 96 NC | 0.5-1.5 yrs pp | ENRICH | Fathers: NS | 50% | ^a^ |
| ^a^ Not enough studies with this outcome measure(s) available to conduct a meta-analysis.  ^b^ Excluded from meta-analyses due to overlapping samples.  ^c^ Excluded from meta-analyses due to lack of data.  All group comparisons not reported in the main results were not significant. DAS, Dyadic Adjustment Scale (Spanier, 1976); DAS-7, Dyadic Adjustment Scale Short Form (Hunsley et al., 2001); ENRICH, Enrich Marital Inventory (Fowers & Olson, 1989); GRIMS, Golombok Rust Inventory of Marital State (Rust et al., 1988; Rust et al., 1990); MMQ, Maudsley Marital Questionnaire (Arrindell et al., 1983); NC, natural conception; NS, not significant; PCI, Primary Communication Inventory (Locke et al., 1990); pp, postpartum; yrs, years. | | | | | | | |

**Supplementary Table S6** Parenting stress

| **Study** | **Country** | **Study groups** | **Time point** | **Outcome measures** | **Main results** | **Quality rating** | **Data included in MA** |
| --- | --- | --- | --- | --- | --- | --- | --- |
| Barnes et al., 2004 | Belgium, Denmark, Greece, Sweden, UK | Mothers: 341 ICSI, 294 IVF, 309 NC  Fathers: 201 ICSI, 190 IVF, 186 NC | 4.5-5.5 yrs pp | PSI-SF | Mothers & fathers: NS | 43% | PSI-SF |
| Colpin & Bossaert, 2008 | Belgium | Mothers: 24 IVF, 20 NC  Fathers: 22 IVF, 16 NC | 15-16 yrs pp | PSI-SF | Mothers & fathers: NS | 57% | ^b^ |
| Colpin & Soenen, 2002 | Belgium | Mothers: 27 IVF, 23 NC  Fathers: 25 IVF, 18 NC | 8-9 yrs pp | PSI | Mothers & fathers: NS | 57% | PSI |
| Cook et al., 1998 | UK | Mothers: 12 IVF, 14 NC  Fathers: 12 IVF, 14 NC  All parents of twins | 4-8 yrs pp | PSI-SF | Mothers & fathers: Higher parenting stress in IVF than NC twin parents | 14% | ^c^ |
| Golombok et al., 1995 | UK | Mothers: 38 IVF, 39 NC  Fathers: 29 IVF, 25 NC | 4-8 yrs pp | PSI-SF | Mothers: NS  Fathers: Less parental distress (PSI-SF subscale) in IVF than NC fathers (own post-hoc t-test) | 43% | PSI-SF |
| Golombok et al., 1996 | UK, Spain, Italy, Netherlands | Mothers: 116 IVF, 120 NC  Fathers: 116 IVF, 120 NC | 4-8 yrs pp | PSI-SF | Mothers: Less parenting stress in IVF (and donor insemination) than NC mothers  Fathers: NS | 29% | ^c^ |
| Hahn & DiPietro, 2001 | Taiwan | Mothers: 53 IVF, 58 NC | 3-7 yrs pp | PSI | Mothers: Less parenting stress in IVF mothers with one child than IVF mothers with more than one child and NC mothers with one or more children | 71% | PSI |
| Knoester et al., 2007 | Netherlands | Parents: 81 ICSI, 76 IVF, 80 NC | 5-8 yrs pp | PSI | Parents: Higher stress due to characteristics of the child (PSI subscale) in ICSI than NC parents | 43% | ^c^ |
| McMahon et al., 2003 | Australia | Mothers: 66 IVF, 46 NC  Fathers: 58 IVF, 36 NC | 5 yrs pp | PSI-SF | Mothers: Higher levels of IVF treatment associated with less parenting stress  Fathers: NS | 50% | ^c^ |
| Ponjaert-Kristoffersen et al., 2004 | Belgium, Sweden, USA | Mothers: 277 ICSI, 212 NC | 4-5 yrs pp | PSI | Mothers: Less parenting stress in ICSI mothers than NC mothers; young ICSI mothers experience less parenting stress than young NC mothers | 43% | PSI |
| Taubman - Ben-Ari et al., 2018 | Israel | Fathers: 76 ART, 96 NC | 0.5-1.5 yrs pp | PSI-SF | Fathers: NS | 50% | PSI-SF |
| van Balen, 1996 | Netherlands | Mothers: 45 IVF, 35 formerly infertile, 35 NC  Fathers 40 IVF, 33 formerly infertile, 28 NC | 2-4 yrs pp | NVOS | Mothers: More parental competence (NVOS subscale) in IVF and formerly infertile mothers than NC mothers  Fathers: NS | 29% | ^a^ |
| ^a^ Not enough studies with this outcome measure(s) available to conduct a meta-analysis.  ^b^ Excluded from meta-analyses due to overlapping samples.  ^c^ Excluded from meta-analyses due to lack of data.  All group comparisons not reported in the main results were not significant. NC, natural conception; NS, not significant; NVOS, Nijmegen Questionnaire Regarding Child-Rearing Circumstances (Robbroeckx & Wels, 1989); pp, postpartum; PSI, Parenting Stress Index (Abidin, 1883; de Brock et al., 1992); PSI-SF, Parenting Stress Index Short Form (Abidin, 1883; 1990; de Brock et al., 1992); yrs, years. | | | | | | | |

**Supplementary Table S7** Parent-child relationship

| **Study** | **Country** | **Study groups** | **Time point** | **Outcome measures** | **Main results** | **Quality rating** | **Data included in MA** |
| --- | --- | --- | --- | --- | --- | --- | --- |
| Barnes et al., 2004 | Belgium, Denmark, Greece, Sweden, UK | Mothers: 225 ICSI, 209 IVF, 210 NC  Fathers: 184 ICSI, 177 IVF, 174 NC  N depends on the variable | 4.5-5.5 yrs pp | PARQ; BAFRT | Mothers: Fewer negative feelings towards their children in ICSI than NC mothers  Fathers: NS | 43% | ^a^ |
| Carson et al., 2010 | UK | Mothers: 99 ART, 198 matched NC, 402 prolonged time to pregnancy, 5556 normal time to pregnancy, 10 574 NC | 3 yrs pp | CPRS | Mothers: NS | 50% | ^a^ |
| Colpin et al., 1995 | Belgium | Mothers: 31 IVF, 31 NC  Fathers: 30 IVF, 29 NC | 2-2.5 yrs pp | RSST; QPAE | Mothers: Employed IVF mothers showed less respect for their children's autonomy and their children showed significantly less persistence and enthusiasm and were significantly more reliant on the mothers’ help compared to non-employed IVF mothers and employed NC mothers  Fathers: NS | 64% | ^a^ |
| Golombok et al., 1995 | UK | Mothers: 41 IVF, 43 NC  Children: 30 IVF, 39 NC | 4-8 yrs pp | Interview with the mother; FRT; Adaptation of SAT | Mothers & fathers (mothers’ report): Greater interaction with their children in IVF parents than NC parents (own post-hoc analysis)  Children: NS | 44% | ^a; b; c^ |
| Golombok et al., 1996 | UK, Spain, Italy, Netherlands | Mothers: 116 IVF, 120 NC  Children: 116 IVF, 120 NC | 4-8 yrs pp | Interview with the mother; FRT | Mothers & fathers (mothers’ report): Greater interaction with their children in IVF (and donor insemination) parents than NC parents  Children: NS | 31% | ^a^ |
| Golombok et al., 2001 | UK | Mothers: 34 IVF, 38 NC  Fathers: 34 IVF, 38 NC  Children: 34 IVF, 38 NC | 12 yrs pp | Interview with the parents; EAI; CTS | Mothers & fathers: NS  Children: Less reasoning during a conflict between IVF father and child than NC father and child (own post-hoc t-test) | 38% | ^b; c^ |
| Golombok et al., 2002 | UK, Spain, Italy, Netherlands | Mothers: 102 IVF, 102 NC  Fathers: 102 IVF, 102 NC  Children: 102 IVF, 102 NC | 11-12 yrs pp | Interview with the parents; EAI; CTS | Mothers & fathers: NS (own post-hoc t-test)  Children: NS | 36% | ^b; c^ |
| Golombok et al., 2009 | UK | Children: 26 IVF, 56 formerly infertile | 17-18 yrs pp | IPPA | Children: NS | 43% | ^a^ |
| Owen & Golombok, 2009 | UK | Mothers: 26 IVF, 63 formerly infertile  Fathers: 26 IVF, 63 formerly infertile | 17-19 yrs pp | Interview with the parents; CBQ | Mothers: Greater disciplinary indulgence in ART than NC mothers  Fathers: NS | 50% | ^a^ |
| Raoul-Duval et al., 1994 | France | Mothers: 29 IVF, 26 NC  N depends on time point | 1.5, 3 yrs pp | Interview; Observation | Mothers: NS | 56% | ^a^ |
| van Balen, 1996 | Netherlands | Mothers 45 IVF, 35 formerly infertile, 35 NC  Fathers 40 IVF, 33 formerly infertile, 28 NC | 2-4 yrs pp | CRPR | Mothers: IVF and formerly infertile mothers experienced more pleasure in their child and reported that they expressed stronger feelings towards the child  Fathers: NS | 29% | ^a^ |
| ^a^ Not enough studies with this outcome measure(s) available to conduct a meta-analysis.  ^b^ Excluded from meta-analyses due to overlapping samples.  ^c^ Excluded from meta-analyses due to lack of data.  All group comparisons not reported in the main results were not significant. BAFRT, Bene-Anthony Family Relations Test (Bene, 1985); CBQ, Conflict Behavior Questionnaire (Prinz et al., 1979); CPRS, Pianta Parent-Child Relationship Scale (Pianta, 1995); CRPR, Child-Rearing Practices Report (Block, 1965; Dekovic, 1991); CTS, Conflict Tactics Scale (Straus, 1979); EAI, Expression of Affection Inventory (Hetherington & Clingempeel, 1992); FRT, Family Relations Test (Bene & Anthony, 1985); IPPA, Inventory of Peer and Parent Attachment (Armsden & Greenberg, 1987); NC, natural conception; NS, not significant; PARQ, Parental Acceptance-Rejection Questionnaire (Rohner, 1999); pp, postpartum; QPAE, Questionnaire for Parental Attitudes and Emotions (Engfer & Schneewind, 1976; Lambermon, 1991); RSST, Rating Scales for Structured Tasks (Erickson et al., 1985); SAT, Separation Anxiety Test (Klagsbrun & Bowlby, 1976); yrs, years. | | | | | | | |

**Supplementary Table S8** Parenting behavior

| **Study** | **Country** | **Study groups** | **Time point** | **Outcome measures** | **Main results** | **Quality rating** | **Data included in MA** |
| --- | --- | --- | --- | --- | --- | --- | --- |
| Barnes et al., 2004 | Belgium, Denmark, Greece, Sweden, UK | Mothers: 302 ICSI, 270 IVF, 275 NC  Fathers: 198 ICSI, 192 IVF, 178 NC  N depends on the variable | 4.5-5.5 yrs pp | Commitment to work and parenting | Mothers: Higher commitment to parenting in ICSI than NC mothers  Fathers: NS | 43% | ^a^ |
| Carson et al., 2010 | UK | Mothers: 99 ART, 198 matched NC, 402 prolonged time to pregnancy, 5556 normal time to pregnancy, 10 574 NC | 3 yrs pp | Daily reading to the child | Mothers: More ART than NC mothers read daily to their children | 50% | ^a^ |
| Colpin & Bossaert, 2008 | Belgium | Mothers: 24 IVF, 20 NC  Fathers: 22 IVF, 16 NC  Children: 24 IVF, 19 NC | 15-16 yrs pp | LAPPS | Mothers: NS  Fathers: NS  Children: NS | 57% | ^a^ |
| Colpin & Soenen, 2002 | Belgium | Mothers: 27 IVF, 23 NC  Fathers: 25 IVF, 18 NC | 8-9 yrs pp | NCRQ; QPG | Mothers: Religion as parenting goal is more important for IVF than NC mothers  Fathers: Adjustment to parents’ expectations as parenting goal is more important for IVF than NC fathers | 57% | ^a^ |
| Cook et al., 1998 | UK | Mothers: 12 IVF, 14 NC  All mothers of twins | 4-8 yrs pp | Interview | Mothers: NS | 19% | ^a^ |
| Egan et al., 2022 | USA | Mothers: 171 ART, 198 NC | ≤5 yrs pp | VCS | Mothers: ART mothers are more likely to perceive their child as vulnerable than NC mothers, especially when a donor sperm and/or egg is used | 50% | ^a^ |
| Fisher et al., 2012 | Australia | Mothers: 153 ART, population-based data | ≤1.5 yrs pp | Risks for residential early parenting service admissions | Mothers: ART mothers are more than three times more likely to be admitted to a residential early parenting service program than mothers in the general population | 29% | ^a^ |
| Golombok et al., 1995 | UK | Mothers: 38 IVF, 39 NC | 4-8 yrs pp | Interview with the mother | Mothers: IVF mothers expressed greater warmth toward their children and showed greater emotional involvement than NC mothers (own post-hoc analysis)  Fathers (mothers’ report): NS | 44% | ^b^ |
| Golombok et al., 2001 | UK | Mothers: 34 IVF, 38 NC  Fathers: 34 IVF, 38 NC  Children: 34 IVF, 38 NC | 12 yrs pp | Interview with the parents; CAFÉ | Mothers: IVF mothers showed lower levels of sensitive responding and were perceived to be more dependable than NC mothers (own post-hoc analysis)  Fathers: NS  Children: NS | 38% | ^b^ |
| Golombok et al., 2002 | UK, Spain, Italy, Netherlands | Mothers: 102 IVF, 102 NC  Fathers: 102 IVF, 102 NC  Children: 102 IVF, 102 NC | 11-12 yrs pp | Interview with the parents; CAFÉ | Mothers & fathers: IVF parents reported greater enjoyment of parenthood and higher emotional involvement than NC parents (own post-hoc analysis)  Children: NS (own post-hoc analysis) | 38% | ^a^ |
| Golombok et al., 2009 | UK | Children: 26 IVF, 56 formerly infertile | 17-18 yrs pp | CAFÉ | Children: NS | 44% | ^b^ |
| Hahn & DiPietro, 2001 | Taiwan | Mothers: 54 IVF, 58 NC  Teachers: 45 IVF, 57 NC  N depends on variable | 3-7 yrs pp | FAPGARI; PPS; CRDS; own items | Mothers: IVF mothers are more protective toward their children, show higher separation anxiety and encouragement of dependency, are more likely to provide their children with preschool enrichment programs, and are less satisfied with aspects of family functioning than NC mothers  Teachers: IVF mothers are more openly affectionate to their child than NC mothers | 63% | ^a^ |
| Owen & Golombok, 2009 | UK | Mothers: 26 IVF, 63 formerly infertile  Fathers: 26 IVF, 63 formerly infertile | 17-19 yrs pp | Interview with the parents; PASAS | Mothers & fathers: NS | 50% | ^a; b^ |
| Pottinger & Palmer, 2013 | Jamaica | Mothers: 19 ART, 55 NC  Fathers: 3 ART, 5 NC | ≤7 yrs pp | IFS; PSQ; own items | Parents: ART parents believe less strongly that a permissive or autocratic parenting style is best for their child and report lower levels of emotional strain or burden from having a child and the child impacting negatively on family life than NC parents | 29% | ^a^ |
| van Balen, 1996 | Netherlands | Mothers 45 IVF, 35 formerly infertile, 35 NC  Fathers 40 IVF, 33 formerly infertile, 28 NC | 2-4 yrs pp | CRPR | Mothers & fathers: NS | 29% | ^a^ |
| ^a^ Not enough studies with this outcome measure(s) available to conduct a meta-analysis.  ^b^ Excluded from meta-analyses due to overlapping samples.  ^c^ Excluded from meta-analyses due to lack of data.  All group comparisons not reported in the main results were not significant. CAFÉ, Child and Adolescent Functioning and Environment Schedule (John & Quinton, 1991); CRDS, Child-Rearing Disagreements Scale (Jouriles et al., 1991); CRPR, Child-Rearing Practices Report (Block, 1965; Dekovic, 1991); FAPGARI, Family APGAR Index (Smilkstein, 1978); IFS, Impact on Family Scale (Stein & Reissman, 1980); LAPPS, Louvain Adolescent Perceived Parenting Scale (Soenens et al., 2004; Beyers and Goossens, 2008); NC, natural conception; NS, not significant; NCRQ, Nijmegen Childrearing Questionnaire (Gerris et al., 1993; Gerrits et al., 1996); PASAS, Parents of Adolescents Separation Anxiety Scale (Hock et al., 2001); pp, postpartum; QPG, Questionnaire Parenting Goals (Gerris et al., 1993); PPS, Parent Protection Scale (Thomasgard et al., 1995); PSQ, Parenting Style Quiz (Active Parenting Publishers); VCS, Vulnerable Child Scale (Perrin et al., 1989); yrs, years. | | | | | | | |

**Supplementary Table S9** Child cognitive development

| **Study** | **Country** | **Study groups** | **Time point** | **Outcome measures** | **Main results** | **Quality rating** | **Data included in MA** |
| --- | --- | --- | --- | --- | --- | --- | --- |
| Al-Hathlol et al., 2020 | Saudi Arabia | 79 IVF, 79 NC | 8-16 yrs old | School performance (parents’ report) | More IVF children received preschool education than NC children | 50% | ^a^ |
| Aoki et al., 2018 | Japan | 270/189 ART, 815/663 NC | 2, 3 yrs old | KIDS (mothers’ report) | At the age of 3 years, better language development in ART than NC children | 36% | ^b^ |
| Aoki et al., 2022 | Japan | Mothers: 189/186 ART, 536/496 NC | 4, 5 yrs old | KIDS (mothers’ report) | Better receptive language development in ART boys at the age of 4 years compared to NC boys | 43% | ^b^ |
| Barbuscia & Mills, 2017 | UK | 214 ART, 15 004 NC | 3, 5, 7, and 11 yrs old | BAS II subscales NV, WR, VS | ART children at ages 3 and 5 years have higher verbal cognitive abilities than NC children but this difference consistently decreases over time and diminishes by age 11; the effect disappeared when controls for parental variables were added | 44% | ^c^ |
| Bay et al., 2013 | Denmark | 14 991 ART, 555 828 NC | 8-17 yrs old | Register data on prevalence of mental retardation and developmental disorders | NS | 71% | ^a^ |
| Berry et al., 2013 | USA | 105 IVF, 292 NC | 3 yrs old | DAS-II; M-HB; NF; A-VF; BRIEF-P | NS | 50% | ^a^ |
| Carson et al., 2010 | UK | 99 ART, 198 matched NC, 402 prolonged time to pregnancy, 5556 normal time to pregnancy, 10 574 NC | 3 yrs old | BAS II subscale NV | Higher verbal abilities in ART than NC children, but this effect can be explained by parental characteristics | 50% | ^b^ |
| Carson et al., 2011 | UK | 96 ART, 6244 NC | 3, 5 yrs old | BAS II subscales NV, PS, PC | Higher verbal abilities in ART than NC children, but this effect can be explained by parental characteristics | 63% | ^b^ |
| Cederblad et al., 1996 | Sweden | 99 IVF, 345 NC (comparison data) | 2-7 yrs old | GMDS | NS | 25% | ^a^ |
| Fallesen, 2023 | Denmark | 11 169 ART, 334 733 NC | 8-11 yrs old | DNT | Higher school performance in ART than NC children before adjustment for covariates and lower performance after adjustment | 57% | ^a^ |
| Farhi et al., 2021 | Israel | 136 ART, 158 NC | 7-9 yrs old | K-BIT-2; K-ABC arithmetic subtest; TEA-ch subscale CC; ROCFT; AaT; TPNEVT | NS | 44% | ^a^; K-BIT-2 |
| Friedlander et al., 2016 | Israel | 67 ART, 67 NC | 1-7 yrs old | MSEL; Vineland (mothers’ report) | Higher communication skills in ART than NC children but comparable communication skills in the crude analysis | 56% | ^a^; Vineland |
| Guo et al., 2024 | China | 60 IVF, 60 NC | 3-6 yrs old | WPPSI; CCDS; GDDS; DDST | Fewer IVF children than NC children show below-average cognitive development | 50% | ^a; c^ |
| Heineman et al., 2019 | Netherlands | 57 ART with ovarian stimulation (OS), 46 ART without OS, 66 subfertile NC | 9 yrs old | WASI; NEPSY-II | NS | 81% | ^a^; WASI |
| Kelly-Vance et al., 2004 | USA | 12 ART (twins), 19 NC (twins) | 2 yrs old | BSID–II subscale MDI | NS | 38% | ^a^ |
| Kennedy et al., 2023 | Australia | 8976 ART, 333 335 NC | 7-9 yrs old | NAPLAN | NS | 63% | ^a^ |
| Knoester et al., 2008 | Netherlands | 86 ICSI, 85 NC | 5-8 yrs old | RAKIT | Lower IQ in ICSI than NC children depending on covariates included | 50% | ^a^ |
| Leslie et al., 2003 | Australia | 97 ICSI, 80 IVF, 110 NC | 5 yrs old | WPPSI-R | NS | 44% | WPPSI-R |
| Leunens et al., 2006 | Belgium | 151 ICSI, 153 NC | 8 yrs old | WISC-R | Slightly higher IQ in ICSI than NC children mainly due to the mothers’ higher education | 38% | WISC-R |
| Leunens et al., 2008 | Belgium | 109 ICSI, 90 NC | 10 yrs old | WISC-R | NS | 38% | ^b^ |
| Levy-Shiff et al., 1998 | Israel | 51 IVF, 51 NC | 9-10 yrs old | WISC-R; VRT; RCT; TRSSA (teacher’s report) | NS | 50% | ^a^; WISC-R |
| Liapi & Polychronopoulou, 2017 | Greece | 40 ICSI, 40 NC | 8-10 yrs old | RPM; ATHINA | NS | 25% | ^a^ |
| Ludwig et al., 2009 | Germany | 276 ICSI, 273 NC | 4-6 yrs old | K-ABC | NS | 50% | K-ABC |
| Luke et al., 2020 | USA | 3311 IVF, 11 944 NC; 3 659 IVF (twins), 746 NC (twins)  N depends on variable | 8-9 yrs old | STAAR | Higher reading and mathematics scores in IVF than NC children | 36% | ^b^ |
| Luke et al., 2021 | USA | 1165 IVF, 3979 NC; 1222 IVF (twins), 257 NC (twins)  N depends on variable | 8-9 yrs old; 10-12 yrs old | STAAR | Higher reading and mathematics scores in IVF than NC children | 36% | ^b^ |
| Mains et al., 2010 | USA | 423 IVF, national norm as comparison data | 8-17 yrs old | ITBS/ITED | Better academic performance in IVF children than national norm; the difference increased from third to ninth grade but decreased from ninth to 11th grade | 21% | ^a^ |
| Norrman et al., 2018 | Sweden | 8323 ART, 1 499 667 NC | 15 yrs old | Total score of school subjects; ninth-grade marks; poor school performance | Higher school performance in ART than NC children before adjustment for covariates and lower performance after adjustment | 57% | ^b^ |
| Norrman et al., 2020 | Sweden | 2571 ICSI, 1 500 709 NC | 8-10 yrs old; 15 yrs old | Total score of school subjects; third- and ninth-grade marks; qualified to enter secondary school; poor school performance | Higher school performance in ART than NC children before adjustment for covariates and lower (third grade) or comparable (ninth grade) performance after adjustment | 64% | ^a; b^ |
| Place & Englert, 2003 | Belgium | 31 ICSI, 19 IVF, 27 NC  N depends on time point | 3, 5 yrs old | WPPSI-R | Lower IQ in ART than NC children before but not after adjustment for covariates | 75% | WPPSI-R |
| Ponjaert-Kristoffersen et al., 2004 | Belgium, Sweden, USA | 296 ICSI, 259 NC | 4-5 yrs old | WPPSI-R | NS | 44% | ^b^ |
| Ponjaert-Kristoffersen et al., 2005 | Belgium, Denmark, Greece, Sweden, UK | 483 ICSI, 399 IVF, 454 NC | 4-6 yrs old | WPPSI-R | NS | 50% | WPPSI-R |
| Pottinger & Palmer, 2013 | Jamaica | 22 ART, 60 NC | ≤7 yrs pp | MSCA; Vineland (parents’ report) | Higher IQ and mean communication scores in ART than NC children | 31% | ^a^; Vineland |
| Punamäki et al., 2016 | Finland | Parents: 255 ART, 278 NC | 6-7 yrs old | FTF (mothers’ and fathers’ report) | NS | 50% | ^a^ |
| Sanchez-Albisua et al., 2011 | Germany | 35 ICSI, 37 NC | 5 yrs old | K-ABC | NS | 38% | K-ABC |
| Sandin et al., 2013 | Sweden | 10 718 ICSI, 19 446 IVF, 2 510 166 NC | 2-27 yrs old | Register data on prevalence of mental retardation | NS | 50% | ^a^ |
| Schendelaar et al., 2016 | Netherlands | 63 ART with ovarian stimulation, 53 ART without ovarian stimulation, 77 subfertile NC | 4 yrs old | K-ABC -II | Negative correlation between time to conceive and IQ | 44% | K-ABC -II |
| Spangmose et al., 2017 | Denmark | 2544 ART, 4985 NC; 1678 ART (twins), 6219 NC (twins) | 15-16 yrs old | General test of academic achievements | Higher academic test scores in ART than NC children before adjustment for covariates and lower (singletons) or comparable (twins) scores after adjustment | 64% | ^a^ |
| Wagenaar et al., 2008b | Netherlands | 233 IVF, 233 subfertile NC | 8-18 yrs old | CITO; extra lessons; repeated school grade; special education; learning or developmental disorders; educational level | NS | 64% | ^a; b^ |
| Wagenaar et al., 2009a | Netherlands | 139 IVF, 143 subfertile NC | 9-18 yrs old | CITO; ANT; TMT | NS | 57% | ^a; b^ |
| Wang et al., 2021 | Sweden | 14 759 ART, 99 103 subfertile NC, 1 422 216 NC | approx. 15-16 yrs old | Ninth-grade marks; eligibility for upper secondary school | Higher school performance in ART than NC children before adjustment for covariates and lower performance after adjustment | 43% | ^a^ |
| Wienecke et al., 2020 | Denmark | 10 099 ART, 5497 subfertile NC, 138 940 NC | approx. 15-16 yrs old | Ninth-grade marks | Higher school performance in ART than NC children before adjustment for covariates and lower performance after adjustment | 71% | ^a^ |
| ^a^ Not enough studies with this outcome measure(s) available to conduct a meta-analysis.  ^b^ Excluded from meta-analyses due to overlapping samples.  ^c^ Excluded from meta-analyses due to lack of data.  All group comparisons not reported in the main results were not significant. AaT, Aleph-ad-Tav (Shani et al., 2006); AEDC, Australian Early Development Census (The Australian Early Development Census, 2020); ANT, Amsterdam Neuropsychological Tasks (De Sonneville, 1999); ATHINA, Athina Test of Learning Difficulties (Paraskevopoulos, 2011); A-VF, action-verb fluency; BAS II, subscales NV/WR/VS, British Ability Scales, subscales naming vocabulary / word reading / verbal similarity (Connelly, 2013); BRIEF-P, Behavior Rating Inventory of Executive Function – Preschool Parent Form (Gioia et al., 1996); BSID-II, subscale MDI, Bayley Scales of Infant Development – second edition, subscale Mental Developmental Index (Bayley, 1993); CCDS, Chinese Child Development Scale (Rong & Houcan, 1994); CITO, national test of educational achievement (Basisonderwijs, 2002); DAS-II, Differential Ability Scales – second edition (Elliot et al., 1997; Elliott, 2007); DDST, Denver Developmental Screening Test (Frankenburg & Dodds, 1967); DNT, Danish National Tests (Beuchert & Nandrup, 2018); FTF, Five to Fifteen (Kadesjö et al., 2004); GDDS, Gesell Developmental Diagnostic Scale (Liu et al., 2019); GMDS, Griffiths Mental Development Scales (Griffiths, 1970); ITBS/ITED, Iowa Tests of Basic Skills / Educational Development (Forsyth et al., 2003; Hoover et al., 1993); K-ABC, Kaufman Assessment Battery for Children (Kaufman & Kaufman, 2001; 2003; 2004); K-BIT-2, Kaufman Brief Intelligence Test (Kaufman & Kaufman, 2004); KIDS, Kinder Infant Development Scale (Miyake et al., 1989); M-HB, modified Hopkins Board (Baron et al., 2010); MSCA, McCarthy Scales of Children’s Abilities (McCarthy, 1972); MSEL, Mullen Scales of Early Learning (Mullen, 1995); NAPLAN, National Assessment Program–Literacy and Numeracy (The Australian Curriculum Assessment and Reporting Authority, 2023); NEPSY (Korkman et al., 2007); NC, natural conception; NF, noun fluency (Baron, 2004; Gaddes & Crockett, 1975); NS, not significant; RAKIT, revised Amsterdam Child Intelligence Test (Bleichrodt et al., 1987); RCT, Reading Comprehension Test (Orthar & Ben Shahar, 1976); ROCFT, Rey-Osterrieth Complex Figure Test (Rey, 1941); RPM, Raven’s Progressive Matrices (Raven et al., 2000); STAAR, State of Texas Assessments of Academic Readiness (Human Resources Research Organization, 2016); TEA-ch, subscale CC, Test of Everyday Attention for Children, subscale Creature Counting (Manly et al., 2001); TMT, Trail Making Test (Reitan, 1979); TPNEVT, Tavor Picture Naming Expressive Vocabulary Test (Tavor, 2011); TRSSA, Rating Scale for School Adjustment (Smilansky & Shephatia, 1976); Vineland, Vineland Adaptive Behaviour Scales (Sparrow et al., 1984; 2005); VRT, Visual Retention Test (Benton,1963); WASI, Wechsler Abbreviated Scale of Intelligence (Wechsler, 1999a); WISC-R, Wechsler Intelligence Scale for Children – revised (Wechsler, 1974; 1976; Vander Steene et al., 1986); WPPSI-R= Wechsler Preschool and Primary Scales of Intelligence – revised (Vander Steene & Bos, 1997; Wechsler, 1989; 1990; 1995; 1999b); yrs, years. | | | | | | | |

**Supplementary Table S10** Child psychomotor development

| **Study** | **Country** | **Study groups** | **Time point** | **Outcome measures** | **Main results** | **Quality rating** | **Data included in MA** |
| --- | --- | --- | --- | --- | --- | --- | --- |
| Al-Hathlol et al., 2020 | Saudi Arabia | 79 IVF, 79 NC | 8-16 yrs old | GMFCS (parents’ report) | NS | 50% | ^a^ |
| Aoki et al., 2018 | Japan | 270/189 ART, 815/663 NC | 2, 3 yrs old | KIDS (mothers’ report) | NS | 36% | ^b^ |
| Aoki et al., 2022 | Japan | Mothers: 189/186 ART, 536/496 NC | 4, 5 yrs old | KIDS (mothers’ report) | NS | 43% | ^b^ |
| Bay et al., 2013 | Denmark | 14 991 ART, 555 828 NC | 8-17 yrs old | Register data on prevalence of developmental disorders | NS | 71% | ^a^ |
| Berry et al., 2013 | USA | 105 IVF, 292 NC | 3 yrs old | Beery VMI; PPT | NS | 50% | ^c^ |
| Farhi et al., 2021 | Israel | 136 ART, 158 NC | 7-9 yrs old | Beery VMI & VP | NS | 44% | Beery VMI & VP |
| Friedlander et al., 2016 | Israel | 67 ART, 67 NC | 1-7 yrs old | MSEL; Vineland (mothers’ report) | Higher motor abilities in ART than NC children but comparable motor abilities in the crude analysis | 56% | ^a^; Vineland |
| Guo et al., 2024 | China | 60 IVF, 60 NC | 3-6 yrs old | CCDS; GDDS | Fewer IVF children than NC children show below-average psychomotor development | 50% | ^a^ |
| Kelly-Vance et al., 2004 | USA | 12 ART (twins), 19 NC (twins) | 2 yrs old | BSID–II | Lower psychomotor development in ART twins than NC twins | 38% | ^a^ |
| Koivurova et al., 2003 | Finland | 150 IVF, 280 NC; 100 IVF (twins), 100 NC (twins) | 1.5, 2, 3 yrs old | Register data on psychomotor development | NS | 29% | ^a^ |
| Leunens et al., 2006 | Belgium | 151 ICSI, 153 NC | 8 yrs old | Movement ABC | NS | 38% | ^b^ |
| Leunens et al., 2008 | Belgium | 109 ICSI, 90 NC | 10 yrs old | Movement ABC | Better balance skills in ICSI than NC children | 38% | ^b^ |
| Levy-Shiff et al., 1998 | Israel | 51 IVF, 51 NC | 9-10 yrs old | VMGT | NS | 50% | ^a^ |
| Ludwig et al., 2009 | Germany | 276 ICSI, 273 NC | 4-6 yrs old | MOT 4–6; Subnormal motor skills (pediatrician report); age at which milestone was reached (parents’ report) | NS | 50% | ^a^ |
| Place & Englert, 2003 | Belgium | 46 ICSI, 32 IVF, 40 NC | 1.5 yrs old | BLS | NS | 75% | BLS |
| Ponjaert-Kristoffersen et al., 2004 | Belgium, Sweden, USA | 181 ICSI, 141 NC | 4-5 yrs old | PDMS | Lower gross and fine motor function in ICSI than NC children but only in the New York population | 44% | ^a^ |
| Ponjaert-Kristoffersen et al., 2005 | Belgium, Denmark, Greece, Sweden, UK | 479 ICSI, 409 IVF, 452 NC | 4-6 yrs old | MSCA Motor Scale | NS | 50% | ^a^ |
| Pottinger & Palmer, 2013 | Jamaica | 22 ART, 60 NC | ≤7 yrs old | Vineland (parents’ report) | Higher motor abilities in ART than NC children | 31% | Vineland |
| Raoul-Duval et al., 1994 | France | 29 IVF, 26 NC  N depends on time point | 1.5, 3 yrs old | BLS | NS | 69% | ^c^ |
| Vo et al., 2021 | Vietnam | 421 ICSI, 421 NC | 0.5-2.5 yrs old | BLS | Slower language and motor coordination development in ICSI than NC children | 75% | BLS |
| Wagenaar et al., 2009a | Netherlands | 139 IVF, 143 subfertile NC | 9-18 yrs old | Beery VMI, VP & MC; ANT; PPT | Lower visual-motor function in IVF than NC children | 50% | ^a^; Beery VMI & VP |
| ^a^ Not enough studies with this outcome measure(s) available to conduct a meta-analysis.  ^b^ Excluded from meta-analyses due to overlapping samples.  ^c^ Excluded from meta-analyses due to lack of data.  All group comparisons not reported in the main results were not significant. ANT, Amsterdam Neuropsychological Tasks (De Sonneville, 1999); Beery VMI/VP/MC, Beery-Buktenica Developmental Test of Visual-Motor Integration / Visual Perception / Motor Coordination (Beery, 1989; Beery & Beery, 2004; Beery & Buktenica, 1989); BLS, Brunet-Lézine Scale (Brunet & Lézine, 1952; 1965); BSID-II, Bayley Scales of Infant Development – second edition (Bayley, 1993); CCDS, Chinese Child Development Scale (Rong & Houcan, 1994); GDDS, Gesell Developmental Diagnostic Scale (Liu et al., 2019); GMFCS, Gross Motor Function Classification System (Palisano et al., 1997); KIDS, Kinder Infant Development Scale (Miyake et al., 1989); MOT, Zimmer/Volkamer Motor Test (Zimmer & Volkamer, 1987); Movement ABC, Movement Assessment Battery for Children (Henderson and Sugden, 1998); MSCA, McCarthy Scales of Children’s Abilities (McCarthy, 1972); MSEL, Mullen Scales of Early Learning (Mullen, 1995); NC, natural conception; NS, not significant; PDMS, Peabody Developmental Motor Scales (Folio & Fewell, 2000); PPT, Purdue Pegboard Test of Manual Dexterity (Gardner & Broman, 1979; Tiffin, 1986); Vineland, Vineland Adaptive Behaviour Scales (Sparrow et al., 1984; 2005); VMGT, Visual Motor Gestalt Test (Bender, 1946); yrs, years. | | | | | | | |

**Supplementary Table S11** Child psychosocial development and mental health

| **Study** | **Country** | **Study groups** | **Time point** | **Outcome measures** | **Main results** | **Quality rating** | **Data included in MA** |
| --- | --- | --- | --- | --- | --- | --- | --- |
| Al-Hathlol et al., 2020 | Saudi Arabia | Parents: 79 IVF, 79 NC | 8-16 yrs old | ADHD, ASD prevalence (parents’ report) | NS | 50% | ^a^ |
| Aoki et al., 2018 | Japan | Mothers: 270/189 ART, 815/663 NC | 2, 3 yrs old | KIDS Social relationships (mothers’ report) | NS | 36% | ^b^ |
| Aoki et al., 2022 | Japan | Mothers: 189/186 ART, 536/496 NC | 4, 5 yrs old | KIDS Social relationships (mothers’ report) | NS | 43% | ^b^ |
| Barbuscia et al., 2019 | UK | Parents: 404 infertility treatment including 180 ART, 12 585 NC | 3, 5, 7, 11, 14 yrs old | SDQ (mostly mothers’ report) | Lower SDQ scores in infertility treatment children than NC children before adjustment for covariates and higher scores after adjustment; the differences decrease with age and become statistically non-significant by age 14; the same analysis with ART children shows a similar pattern | 50% | SDQ (only of ART and NC children) |
| Barnes et al., 2004 | Belgium, Denmark, Greece, Sweden, UK | Mothers: 345 ICSI, 301 IVF, 310 NC  Fathers: 193 ICSI, 191 IVF, 175 NC | 4.5-5.5 yrs old | CBCL (mothers’ and fathers’ report) | NS | 43% | CBCL |
| Bay et al., 2013 | Denmark | Children: 14 991 ART, 555 828 NC | 8-17 yrs old | Register data on prevalence of mental disorders | Higher risk of tic disorders in ART than NC children | 71% | ^a^ |
| Berry et al., 2013 | USA | Mothers: 105 IVF, 292 NC | 3 yrs old | BASC‐2 (mothers’ report) | NS | 36% | ^a^ |
| Carson et al., 2013 | UK | Mothers: 101 ART, 6 312 NC | 5, 7 yrs old | SDQ (mothers’ report) | Higher SDQ scores in ART than NC children | 57% | ^b^ |
| Cederblad et al., 1996 | Sweden | Mothers: 99 IVF, 141 NC (comparison data)  N depends on variable | 2-7 yrs old | CBCL (mothers’ report); Interview about the child's behavior (mothers’ report); ADHD assessment (child psychiatrist) | Less disturbed behavior in IVF than NC children | 21% | ^a; c^ |
| Colpin & Bossaert, 2008 | Belgium | Mothers: 24 IVF, 20 NC  Fathers: 22 IVF, 16 NC  Children: 24 IVF, 19 NC | 15-16 yrs old | CBCL (mothers’ and fathers’ report); YSR (self-report) | NS | 57% | ^b^; YSR |
| Colpin & Soenen, 2002 | Belgium | Mothers: 27 IVF, 23 NC  Fathers: 23 IVF, 16 NC  Teachers: 17 IVF, 11 NC | 8-9 yrs old | CBCL (mothers’ and fathers’ report); TRF (teacher’s report) | NS | 57% | CBCL; TRF |
| Cook et al., 1998 | UK | Mothers: 12 IVF (twins), 14 NC (twins)  Teachers: 12 IVF (twins), 14 NC (twins) | 4-8 yrs old | Rutter A (mothers’ report) & B Scale (teacher’s report) | NS | 14% | ^c^ |
| Davidovitch et al., 2018 | Israel | 108 548 all conception modes | 5-14 yrs old | Register data on prevalence of ASD | NS | 36% | ^a^ |
| Diop et al., 2019 | USA | 10 147 ART, 441 898 NC | ≤3 yrs old | Register data on prevalence of ASD | NS | 64% | ^a^ |
| Eisemann et al., 2023 | Germany | Children: 521 ICSI; 418 NC | 14-18 yrs old | SDQ; KINDL (all child’s report) | Higher KINDL scores in ICSI than NC children | 36% | ^a^; SDQ |
| Fountain et al., 2015 | USA | 48 865 ART, 5 877 386 NC | 7-17 yrs old | Register data on prevalence of ASD | Higher risk of ASD in ART than NC children | 57% | ^a^ |
| Friedlander et al., 2016 | Israel | 67 ART, 67 NC | 1-7 yrs old | ADOS; Vineland (mothers’ report) | NS | 56% | ^a^; Vineland |
| Golombok et al., 1990 | UK | 26 IVF, comparison data | 2-5 yrs old | PBCL | Higher incidence of behavioral and emotional problems in IVF than NC children | 13% | ^a^ |
| Golombok et al., 1995 | UK | Mothers: 41 IVF, 43 NC  Teachers: 28 IVF, 34 NC | 4-8 yrs old | Interview about child’s psychiatric disorder (mothers’ report); Rutter A (mothers’ report) & B Scale (teachers’ report) | NS | 43% | ^a^ |
| Golombok et al., 1996 | UK, Spain, Italy, Netherlands | Mothers: 116 IVF, 120 NC  Teaches: 116 IVF, 120 NC | 4-8 yrs old | Rutter A (mothers’ report) & B Scale (teachers’ report) | NS | 29% | ^b; c^ |
| Golombok et al., 2001 | UK | Mothers: 34 IVF, 38 NC  Children: 34 IVF, 38 NC  Teachers: 34 IVF, 38 NC | 12 yrs old | Interview about child’s psychiatric disorder (mothers’ report); SDQ (mothers’ and teachers’ report); SAICA (child’s report); CAFÉ (child’s report) | NS | 36% | ^a; b; c^ |
| Golombok et al., 2002 | UK, Spain, Italy, Netherlands | Mothers: 102 IVF, 102 NC  Children: 102 IVF, 102 NC  Teachers: 102 IVF, 102 NC | 11-12 yrs old | SDQ (mothers’ and teachers’ report); CAFÉ (child’s report) | IVF children are less likely to engage in physical aggression towards peers than NC children (own post-hoc analysis) | 36% | ^b^; SDQ |
| Golombok et al., 2009 | UK | Children: 26 IVF, 56 formerly infertile NC | 17-18 yrs old | SCL-90-R (child’s report); CAFÉ (child’s report) | Greater confidence in peer relationships among IVF than NC children | 43% | ^a; b^ |
| Guo et al., 2024 | China | 60 IVF, 60 NC | 3-6 yrs old | GDDS | NS | 31% | ^a^ |
| Hahn & DiPietro, 2001 | Taiwan | Mothers: 54 IVF, 58 NC  Teachers: 45 IVF, 57 NC  N depends on variable | 3-7 yrs old | ECBI (mothers’ report); PBCL (teachers’ report); SESBI (teachers’ report); own items (teachers’ report) | Better compliance to set limits in IVF than NC children | 71% | ^a^; ECBI |
| Hammarberg et al., 2023 | Australia | Children: 193 ART, 86 NC | 22-35 yrs old | WHOQoL-BREF | Better quality of life in ART than NC children | 43% | ^a^ |
| Heineman et al., 2019 | Netherlands | Parents: 57 ART with ovarian stimulation, 46 ART without OS, 66 subfertile NC  Teachers: 57 ART with ovarian stimulation, 46 ART without OS, 66 subfertile NC | 9 yrs old | CBCL (parents’ report); TRF (teachers’ report) | NS | 71% | ^b^; TRF |
| Hvidtjørn et al., 2011 | Denmark | 14 991 IVF, 555 828 NC | 7-15 yrs old | Register data on prevalence of ASD | Higher risk of ASD in ART than NC children before but not after adjusting for covariates | 43% | ^a^ |
| Källén et al., 2011 | Sweden | Children: 28 158 IVF, 2 417 886 NC | ≤28 yrs old | Register data on prevalence of drug-treated ADHD | Higher risk of drug-treated ADHD in IVF than NC children before but not after adjustment for infertility duration | 50% | ^a^ |
| Kennedy et al., 2023 | Australia | 4697 ART, 168 503 NC | 4-6 yrs old | AEDC | NS | 63% | ^a^ |
| Klausen et al., 2017 | Denmark | Children: 858 ART, 3 436 NC  N depends on variable | 3, 7, 14, 18 yrs old | CBCL (at age 14; mothers’ report); register data on prevalence of mental disorders | Lower CBCL scores in ART than NC children | 50% | ^a^; CBCL |
| Knoester et al., 2007 | Netherlands | Parents: 81 ICSI, 76 IVF, 80 NC | 5-8 yrs old | CBCL (parents’ report); Prevalence of mental disorders (parents’ report) | Higher prevalence of ASD in ICSI than NC children | 43% | ^a; c^ |
| Lehti et al., 2013 | Finland | Children: 229 IVF, 16 352 NC | 2-16 yrs old | Register data on prevalence of ASD | NS | 57% | ^a^ |
| Levy-Shiff et al., 1998 | Israel | Parents: 51 IVF, 51 NC  Children: 51 IVF, 51 NC  Teachers: 51 IVF, 51 NC | 9-10 yrs old | CSQ (teachers’ report); STAI (child report); CDI (child report); CAS (child report); CBI (parents’ report); TRSSA (teachers’ report) | Poorer socioemotional adjustment to school and higher anxiety, aggression, and depression in IVF than NC children | 36% | ^a^ |
| Liapi & Polychronopoulou, 2017 | Greece | Parents: 40 ICSI, 40 NC | 8-10 yrs old | CBCL (mostly mothers’ report) | Activities and school subscale scores are in favor of the ICSI children | 21% | CBCL |
| Montgomery et al., 1999 | USA | Parents: 494 IVF, comparison data  Children: 494 IVF, comparison data  Teachers: 494 IVF, comparison data | 4-18 yrs old | CBCL (parents’ report); YSR (child report); TRF (teachers’ report) | NS | 21% | ^c^ |
| Özbaran et al., 2013 | Turkey | Mothers: 35 ART, 35 NC | 2-13 yrs old | K-SADS (children’s & parents’ report); CBCL (mothers’ report) | Higher prevalence of feeding disorders in ART than NC children; higher Internalizing and Externalizing problems subscale scores on CBCL in ART than NC children | 36% | ^a^; CBCL |
| Ponjaert-Kristoffersen et al., 2004 | Belgium, Sweden, USA | Mothers: 265 ICSI, 206 NC | 4-5 yrs old | CBCL (mothers’ report) | Lower CBCL scores in ICSI than NC children in the CNY subsample | 43% | CBCL |
| Pottinger & Palmer, 2013 | Jamaica | Parents: 22 ART, 60 NC | ≤7 yrs pp | Vineland (parents’ report) | Higher mean socialization scores in ART than NC children | 25% | Vineland |
| Punamäki et al., 2016 | Finland | Parents: 255 ART, 278 NC | 6-7 yrs old | BASC (mothers’ and fathers’ report); SSRS (mothers and fathers’ report); CBS (mothers’ and fathers’ report) | NS | 50% | ^a^ |
| Rissanen et al., 2020 | Finland | Children: 17 610 ART, 1 368 346 NC | ≤22 yrs old | Register data on prevalence of mental disorders | Fewer mental disorder diagnoses before but more after adjusting for covariates in ART than NC children; ART children receive the diagnoses at a younger age than NC children | 57% | ^a^ |
| Sanchez-Albisua et al., 2011 | Germany | Parents: 35 ICSI, 37 NC | 5 yrs old | VBV (parents’ report) | NS | 36% | ^a^ |
| Sandin et al., 2013 | Sweden | Children: 10 718 ICSI, 19 446 IVF, 2 510 166 NC | 2-27 yrs old | Register data on prevalence of ASD | NS | 50% | ^a^ |
| Schendelaar et al., 2016 | Netherlands | Parents: 63 ART with ovarian stimulation, 53 ART without ovarian stimulation, 77 subfertile NC | 4 yrs old | CBCL (parents’ report) | NS | 43% | CBCL |
| Schieve et al., 2015 | USA | Children: 530 ART, 29 953 NC | ≤14 yrs old | Register data on diagnosis age and symptom severity of ASD | Lower diagnosis age and symptom severity in ART than NC children before but not after controlling for covariates | 50% | ^a^ |
| Shelton et al., 2009 | UK, USA | Mothers: 735 IVF, comparison data  Fathers: 513, comparison data | 5-9 yrs old | SDQ (mothers’ and fathers’ report) | Lower conduct problems and prosocial behavior among IVF than NC children according to fathers’ report | 21% | SDQ |
| Sutcliffe et al., 2004 | UK | Parents: 151 ICSI, 112 NC | 2 yrs old | ECBI (parents’ report) | Fewer behavioral problems in ICSI children than NC children | 36% | ECBI |
| van Balen, 1996 | Netherlands | Mothers: 45 IVF, 35 formerly infertile, 35 NC  Fathers: 40 IVF, 33 formerly infertile, 28 NC | 2-4 yrs old | Behavior scale (mothers’ and fathers’ report) | The IVF children were characterized as more social and less obstinate by their mothers but not by their fathers | 21% | ^a^ |
| Wagenaar et al., 2009b | Netherlands | Parents: 130 IVF, 142 formerly infertile NC  Teachers: 75 IVF, 82 NC | 9-18 yrs old | CBCL (mostly mothers’ report); TRF (teachers’ report) | Lower CBCL scores in IVF than NC children | 57% | CBCL; TRF |
| Wagenaar et al., 2011 | Netherlands | Children; 86 IVF, 97 NC formerly infertile NC | 11-18 yrs old | YSR (child report) | NS | 50% | YSR |
| Wang et al., 2021 | Sweden | Children: 14 759 ART, 99 103 subfertile NC, 1 422 216 NC | 15 yrs old | Register data on prevalence of ADHD | Lower risk of ADHD in ART than NC children before but not after controlling for covariates | 43% | ^a^ |
| Wang et al., 2022 | Sweden | Children: 31 565 ART, 95 558 subfertile NC, 1 094 689 NC | ≤25 yrs old | Register data on prevalence of mental disorders | Higher risk of OCD in ART than NC children before but not after controlling for covariates | 43% | ^a^ |
| Wijs et al., 2022 | Australia | Parents: 150/160 ART, 1781 NC/1351 NC  Children: 151/161 ART, 1563 NC/1232 NC | 14, 17 yrs old | CBCL (parents’ report); YSR (child’s report); BDI-Y (child’s report); children’s diagnosis (parents’ report) | ART children show fewer self-reported externalizing problems, more parent-reported internalizing problems, and higher anxiety risk than NC children; At the age of 14, there is also a higher prevalence of depression in ART children compared to NC children | 29% | ^a^; CBCL; YSR |
| Winter et al., 2024 | USA | Children: 95 149 ART, 95 149 NC | 0-19 yrs old | Register data on prevalence of ASD | Higher risk of ASD in ART than NC children | 57% | ^a^ |
| Yeung et al., 2023 | USA | Mothers: 1239 NC, 375 ART | 7-11 yrs old | CBCL; VADPRS; children’s diagnosis (all mothers’ report) | Higher risk of anxiety or depression in ART than NC children before and after adjustment for parental variables | 71% | ^a^ |
| ^a^ Not enough studies with this outcome measure(s) available to conduct a meta-analysis.  ^b^ Excluded from meta-analyses due to overlapping samples.  ^c^ Excluded from meta-analyses due to lack of data.  All group comparisons not reported in the main results were not significant. AEDC, Australian Early Developmental Census (The Australian Early Development Census, 2020); ADHD, attention deficit hyperactivity disorder; ADOS, Autism Diagnostic Observation Schedule (Gotham et al., 2007); ASD, autism spectrum disorder; BASC(-2), Behavioral Assessment Scales for Children(-2) (Reynolds & Kamphaus, 1992); BDI-Y, Beck Depression Inventory for Youth (Beck et al., 2001); CAFÉ, Child and Adolescent Functioning and Environment Schedule (John & Quinton, 1991); CAI, Children's Aggression Inventory (Feshbach, 1966); CBCL, Child Behavior Checklist (Achenbach, 1991a; Achenbach & Edelbrock, 1983; Achenbach & Rescorla, 2000; 2001; Erol et al., 1995; Roussou, 2003; Verhulst et al., 1996; 2000); CBI, Child's Behavior Inventory (Shepherd et al., 1971); CBS, Child Behaviour Scale (Rutter, 1967); CDI, Children's Depression Inventory (Kovacs, 1978; Kovacs & Beck, 1977); CSQ, Conners Symptoms Questionnaire (Conners, 1973); ECBI, Eyberg Child Behavior Inventory (Eyberg, 1974; 1978); GDDS, Gesell Developmental Diagnostic Scale (Liu et al., 2019); KIDS, Kinder Infant Development Scale (Miyake et al., 1989); KINDL, German generic quality of life instrument for children (Ravens-Sieberer & Bullinger, 1998); K-SADS, Kiddie-Schedule for Affective Disorders and Schizophrenia (Gokler et al., 2004; Kaufman et al., 1997); NC, natural conception; NS, not significant; PBCL, Pre-School Behaviour Checklist (McGuire & Richman, 1988; 1989); SAICA, Social Adjustment Inventory for Children and Adolescents (John et al., 1987); SCL-90-R, Symptom Checklist-90-R (Derogatis, 1994); SDQ, Strengths and Difficulties Questionnaire (Goodman, 1994; 1997; 2001); SESBI, Sutter-Eyberg Student Behavior Inventory (Sutter & Eyberg, 1984); SSRS, Social Skills Rating System (Gresham & Elliot, 1990); STAI, State-Trait Anxiety Inventory for Children (Spielberger,1973); TRF, Teacher’s Report Form (Achenbach, 1991b; Achenbach & Rescorla, 2001; Verhulst et al., 1997a); TRSSA, Rating Scale for School Adjustment (Smilansky & Shephatia, 1976); VADPRS, Vanderbilt ADHD Diagnostic Parent Rating Scale (Bard et al., 2013); VBV, German Behavioral Questionnaire for Preschoolers (Döpfner et al., 1993); Vineland, Vineland Adaptive Behaviour Scales (Sparrow et al., 1984; 2005); WHOQoL-BREF, World Health Organization Quality of Life – Brief assessment (WHOQUAL Group, 1998); yrs, years; YSR, Youth Self-Report (Achenbach, 1991c; Verhulst et al., 1997b). | | | | | | | |

**References of the outcome measures**

Abidin RR. Parenting Stress Index (PSI) Manual. Charlottesville, VA: Pediatric Psychology Press; 1983.

Abidin RR. Parenting Stress Index short form – test manual. Charlottesville, VA: Pediatric Psychology Press; 1990.

Achenbach TM. Manual for the Child Behavior Checklist/4-18 and 1991 profile. Burlington, VT: University of Vermont, Department of Psychiatry; 1991a.

Achenbach TM. Manual for the Teacher’s Report Form. Burlington, VT: University of Vermont, Department of Psychiatry; 1991b.

Achenbach TM. Manual for the Youth Self-Report and 1991 Profiles. Burlington, VT: University of Vermont, Department of Psychiatry; 1991c.

Achenbach TM, Edelbrock C. Manual for the Child Behavior Checklist and Revised Child Behavior Profile. Burlington, VT: University of Vermont, Department of Psychiatry; 1983.

Achenbach TM, Rescorla LA. Manual for the ASEBA Preschool Forms & Profiles. Burlington, VT: University of Vermont, Department of Psychiatry; 2000.

Achenbach TM, Rescorla LA. Manual for the ASEBA school-age forms & profiles: An integrated system of multi-informant assessment. Burlington, VT: University of Vermont, Department of Psychiatry; 2001.

Active Parenting. Parenting Quiz. Accessed Nov 9 2024. <https://activeparenting.com/for-parents/parenting-quiz/>.

Armsden GC, Greenberg MT. The Inventory of Parent and Peer Attachment: Individual differences and their relationship to psychological well-being in adolescence. Journal of Youth and Adolescence. 1987;16:427–54. https://doi.org/10.1007/BF02202939.

Arrindell WA, Boelens W, Lambert H. On the psychometric properties of the Maudsley Marital Questionnaire (MMQ): Evaluation of self-ratings in distressed and ‘normal’ volunteer couples based on the Dutch version. Pers Individ Dif. 1983;4:293–306. https://doi.org/10.1016/0191-8869(83)90151-4.

Bard DE, Wolraich ML, Neas B, Doffing M, Beck L. The psychometric properties of the Vanderbilt attention-deficit hyperactivity disorder diagnostic parent rating scale in a community population. J Dev Behav Pediatr. 2013;34:72–82. https://doi.org/10.1097/DBP.0b013e31827a3a22.

Baron IS. Neuropsychological evaluation of the child. Oxford: Oxford University Press; 2004.

Baron IS, Erickson K, Ahronovich MD, Litman FR, Brandt J. Spatial location memory discriminates children born at extremely low birth weight and late-preterm at age three. Neuropsychology. 2010;24:787–94. https://doi.org/10.1037/a0020382.

Basisonderwijs E. CITO. Arnhem: Citogroep; 2002.

Bayley N. Bayley Scales of Infant Development. 2nd edn. New York: Psychological Corporation; 1993.

Beck JS, Beck AT, Jolly JB. Beck Youth Inventories of Emotional and Social Impairment Manual. New York: Psychological Corporation; 2001.

Beck A, Steer R. The Beck Depression Inventory Manual. New York: Psychological Corporation; 1987.

Beck AT, Ward CH, Mendelson M, Mock J, Erbaugh J. An inventory for measuring depression. Arch Gen Psychiatry. 1961;4:561–71. https://doi.org/10.1001/archpsyc.1961.01710120031004.

Beery KE. Developmental Tests of Visual-motor Integration with Supplemented Developmental tests of Visual Perception and Motor Coordination. Modern Curriculum Press; 1989.

Beery KE, Beery NA. The Beery-Buktenica Developmental Test of Visual-Motor Integration: administration, scoring, and teaching manual. 5th edn. London: NCS Pearson; 2004.

Beery KE, Buktenica NA. Developmental test of Visual-motor integration: Administration, Scoring and Teaching Manual. Modern Curriculum Press; 1989.

Bender L. Instuctions for the use of the Visual Motor Gestalt Test. Chicago: American Orthopsychiatry Association; 1946.

Bene E. Manual for the Family Relations Test. 2nd edn. Slough: NFER; 1985.

Bene E, Anthony J. Manual for the Family Relations Test. Slough: NFER; 1985.

Benton AL. The revised Visual Retention Test. 3rd edn. New York: Psychological Corporation; 1963.

Beuchert LV, Nandrup AB. The Danish National Tests at a Glance. Natl Tidsskr. 2018.

Bleichrodt N, Resing WCM, Drenth PJD, Zaal JN. Intelligentie-meting bij kinderen. Leiden: Swets & Zeitlinger; 1987.

Brunet O, Lezine I. Le developpement psychologique de la premiere enfance. EAP; 1952.

Brunet O, Lézine I. Le développement psychologique de la petite enfance. PUF; 1965.

Connelly R. Millennium Cohort Study Data Note: Interpreting Test Scores. London: Centre for Longitudinal Studies; 2013.

Conners CK. Rating scale for use in drug studies with children. Psychopharmacol Bull. 1973;12:24–84.

Craig AR, Franklin JA, Andrews G. A scale to measure locus of control of behaviour. Br J Med Psychol. 1984;57:173–80. https://doi.org/10.1111/j.2044-8341.1984.tb01597.x.

De Brock AJLL, Vermulst AA, Gerris JRM, Abidin RR. Nijmeegse Ouderlijke Stress Index (NOSI). Leiden: Swets & Zeitlinger; 1992.

Derogatis LR. SCL-90-R symptoms check list: administration, scoring and procedures manual. Minneapolis: National Computer Systems; 1994.

De Sonneville LMJ. Amsterdam neuropsychological tasks: a computer-aided assessment program. In: Den Brinker BPMLM, Beek PJ, Brand AN, Maarse SJ, Mulder LJM, editors. Cognitive Ergonomics, Clinical Assessment and Computer-assisted Learning: Computers in Psychology. Leiden: Swets & Zeitlinger; 1999. pp. 187–203.

Diener E, Emmons RA, Larsen RJ, Griffin S. The Satisfaction With Life Scale. J Pers Assess. 1985;49:71–5. https://doi.org/10.1207/s15327752jpa4901_13.

Döpfner M, Berner W, Fleischmann T, Schmidt M. Verhaltensbeurteilungsbogen für Vorschulkinder (VBV 3–6). Weinheim: Beltz; 1993.

Elliott CD. Differential Ability Scales eII. Harcourt Assessment; 2007.

Elliott CD, Smith P, McCulloch K. British ability scales. 2nd ed (BAS II): technical manual. Slough: NFER; 1997.

Engfer A, Schneewind KA. Ein Fragebogen selbstperzipiertes elterlichen Erziehungseinstellungen (unpublished manuscript). Trier: Trier University; 1976.

Erickson MF, Sroufe LA, Egeland B. The Relationship between Quality of Attachment and Behavior Problems in Preschool in a High-Risk Sample. Monographs of the Society for Research in Child Development. 1985;50:147. https://doi.org/10.2307/3333831.

Erol N, Arslan BL, Akçakın M. The adaptation and standardization of the Child Behavior Checklist among 6-18-year-old Turkish children. In: Sergeant J, editor: Eunethydis: European Approaches to Hyperkinetic Disorder. Fotoratar; 1995. pp. 97–114.

Eyberg SM. Eyberg Child Behavior Inventory. Lake Magdalene: Psychological Assessment Resources; 1974.

Eyberg SM, Ross AW. Assessment of child behavior problems: The validation of a new inventory. J Clin Child Psychol. 1978;7:113–6.

Fahrenberg J, Myrtek M, Schumacher J, Brähler E. Fragebogen zur Lebenszufriedenheit (FLZ). Göttingen: Hogrefe Verlag für Psychologie; 2000.

Feshbach S. Child Aggression Scale. California: University of California; 1966.

Fliege H, Rose M, Arck P, Levenstein S, Klapp BF. Validierung des "Percived Stress Questionnaire" (PSQ) an einer deutschen Stichprobe. Diagnostica. 2001;47:142–52. doi:10.1026//0012-1924.47.3.142.

Folio MR, Fewell RR. Peabody Developmental Motor Scales. 2nd ed. Indianapolis: PRO-ED; 2000.

Forsyth RA, Ansley TN, Feldt LS, Alnot SD. Iowa Tests of Educational Development guide to research and development. Itasca, IL: Riverside Publishing; 2003.

Fowers BJ, Olson DH. Enrich marital inventory: A discriminant validity and cross-validation assessment. J Marital Fam Ther. 1989;15:65–79. https://doi.org/10.1111/j.1752-0606.1989.tb00777.x.

Frankenburg WK, Dodds JB. The Denver developmental screening test. The Journal of pediatrics. 1967.

Gaddes WH, Crockett DJ. The Spreen-Benton aphasia tests, normative data as a measure of normal language development. Brain Lang. 1975;2:257–80. https://doi.org/10.1016/s0093-934x(75)80070-8.

Gardner RA, Broman M. The Purdue pegboard: Normative data on 1334 school children. J Clin Child Psychol. 1979;8:156–62. https://doi.org/10.1080/15374417909532912.

Gerris JRM. Parenting in Dutch families: A representative description of Dutch family life in terms of validated concepts representing characteristics of parents, children, the family as a system and parental socio-cultural value orientations. Nijmegen: Institute of Family Studies, University of Nijmegen; 1993.

Gerrits LAW, Dekovic M, Groenendaal JHA. Parenting behavior. In: Rispens J, Hermanns JMA, Meeus WHJ, editors. Childrearing in the Netherlands. Van Gorcum; 1996. pp. 41–69.

Gioia G, Espy K, Isquith P. Behavior Rating Inventory of Executive Function Preschool Version. Lake Magdalene: Psychological Assessment Resources; 1996.

Gokler B, Unal F, Pehlivanturk F, Kültür EC, Akdemir D, Taner Y. Reliability and validity of schedule for affective disorders and schizophrenia for school age children-present and lifetime version – Turkish version (K-SADS-PL-T). Turk J Child Adolesc Mental Health. 2004;11:109–16.

Goldberg DP, Hillier VF. A scaled version of the General Health Questionnaire. Psychol Med. 1979;9:139–45. https://doi.org/10.1017/s0033291700021644.

Goodman R. A modified version of the Rutter parent questionnaire including extra items on children's strengths: a research note. J Child Psychol Psychiatry. 1994;35:1483–94. https://doi.org/10.1111/j.1469-7610.1994.tb01289.x.

Goodman R. The Strengths and Difficulties Questionnaire: a research note. J Child Psychol Psychiatry. 1997;38:581–6. https://doi.org/10.1111/j.1469-7610.1997.tb01545.x.

Goodman R. Psychometric properties of the strengths and difficulties questionnaire. J Am Acad Child Adolesc Psychiatry. 2001;40:1337–45. https://doi.org/10.1097/00004583-200111000-00015.

Gotham K, Risi S, Pickles A, Lord C. The Autism Diagnostic Observation Schedule: revised algorithms for improved diagnostic validity. J Autism Dev Disord. 2007;37:613–27. https://doi.org/10.1007/s10803-006-0280-1.

Gresham FM, Elliot SN. Social Skills Rating System. Manual. Circle Pines, MN: American Guidance Service; 1990.

Griffiths R. The abilities of young children: A comprehensive system of mental measurement for the first eight years of life. London: Child Development Research Centre; 1970.

Henderson SE, Sugden DA. Movement Assessment Battery for Children. Manual. Leiden: Swets & Zeitlinger B.V; 1998.

Hetherington EM, Clingempeel WG. Coping with marital transitions: A family systems perspective. Monogr Soc Res Child Dev. 1992;57:1–242. https://doi.org/10.2307/1166050.

Hisli N. Beck Depresyon Envanterinin Gecerligi Uzerine bir Calisma. J Psychol. 1988;6:118–22.

Hock E, Eberly M, Bartle-Haring S, Ellwanger P, Widaman KF. Separation anxiety in parents of adolescents: theoretical significance and scale development. Child Dev. 2001;72:284–98. https://doi.org/10.1111/1467-8624.00279.

Hoover HD, Hieronymus AN, Frisbie DA, Dunbar SB. Iowa Tests of Basic Skills complete battery Form K. Chicago, Riverside; 1993.

Human Resources Research Organization. Wechsler preschool and primary scales of intelligence. Independent evaluation of the validity and reliability of STAAR grades 3-8 assessment scores: part 2. 2016. <https://tea.texas.gov/student-assessment/reports-and-studies/independent-evaluation-of-the-validity-and-reliability-of-staar-grades-3-8-assessmentspart2.pdf>. Accessed 15 Nov 2024.

Hunsley J, Best M, Lefebvre M, Vito D. The seven-item short form of the Dyadic Adjustment Scale: Further evidence for construct validity. Am J Fam Ther. 2001;29:325–35. https://doi.org/10.1080/01926180152588734.

John K, Gammon GD, Prusoff BA, Warner V. The Social Adjustment Inventory for Children and Adolescents (SAICA): testing of a new semistructured interview. J Am Acad Child Adolesc Psychiatry. 1987;26:898–911. https://doi.org/10.1097/00004583-198726060-00015.

John K, Quinton D. Child and Adolescent Functioning and Environment Schedule (Revised). London: MRC Child Psychiatry Unit; 1991.

Jouriles EN, Murphy CM, Farris AM, Smith DA, Richters JE, Waters E. Marital adjustment, parental disagreements about child rearing, and behavior problems in boys: Increasing the specificity of the marital assessment. Child Dev. 1991;62:1424–33. https://doi.org/10.2307/1130816.

Kadesjö B, Janols LO, Korkman M, Mickelsson K, Strand G, Trillingsgaard A, Gillberg C. The FTF (Five to Fifteen): the development of a parent questionnaire for the assessment of ADHD and comorbid conditions. Eur Child Adolesc Psychiatry. 2004;13:3–13. https://doi.org/10.1007/s00787-004-3002-2.

Kaufman J, Birmaher B, Brent D, Rao U, Flynn C, Moreci P, Williamson D, Ryan N. Schedule for Affective Disorders and Schizophrenia for School-Age Children-Present and Lifetime Version (K-SADS-PL): Initial reliability and validity data. J Am Acad Child Adolesc Psychiatry. 1997;36:980–8. https://doi.org/10.1097/00004583-199707000-00021.

Kaufman AS, Kaufman NL. Kaufman Assessment Battery for Children (K-ABC). Göttingen: Hogrefe; 2001.

Kaufman AS, Kaufman NL. Kaufman Assessment Battery for Children (KABC-II). 2nd ed. Circle Pines, MN: American Guidance Service; 2003.

Kaufman AS, Kaufman NL. Kaufman Brief Intelligence Test. 2nd ed. Circle Pines, MN: American Guidance Service; 2004.

Klagsbrun M, Bowlby J. Responses to separation from parents: A clinical test for young children. British Journal of Projective Psychology & Personality Study. 1976;21:7–27.

Korkman M, Kirk U, Kemp SL. Clinical NEPSY-II, Manual I. New York: Psychological Corporation; 2007.

Kovacs M. Rating scales to assess depression in school-aged children. Acta Paedopsychiatr. 1978;46:305–15.

Kovacs M, Beck AT. An empirical clinical approach toward a definition of childhood depression. In: Schutterbrand J, Raskin A, editors. Depression in childhood: Diagnosis, treatment, and conceptual models. Rockville: National Institute of Mental Health; 1977. pp. 1–25.

Lambermon MWE. Video of folder? Korte- en lange termijneffecten van voorlichting over vroegkinderlijke opvoeding [Doctoral dissertation]. Leiden: Leiden State University; 1991.

Liu ZM, Wei RM, Cai KL, Wang JG, Chen AG. Application and study of the Gesell developmental diagnostic scale on 1568 children with autism spectrum disorders and developmental language disorders. China Academic Journal Electronic Publishing House. 2019:2329–30.

Locke HJ, Sabaght F, Thomes MM. Primary Communication Inventory (PCI). In: Fisher J, Corcoran K, editors. Measures for clinical practice: A source book. Vol. 1. New York: Free Press; 1990. pp. 166–9.

Manly T, Anderson V, Nimmo-Smith I, Turner A, Watson P, Robertson IH. The differential assessment of children's attention: the Test of Everyday Attention for Children (TEA-Ch), normative sample and ADHD performance. J Child Psychol Psychiatry. 2001;42:1065–81. https://doi.org/10.1111/1469-7610.00806.

McCarthy DA. Manual for the McCarthy Scales of Children’s Abilities. New York: Psychological Corporation; 1972.

McGuire J, Richman N. The Pre-School Behaviour Checklist. Slough: NFER-Nelson; 1988.

McGuire J, Richman N. Pre-School Behavior Checklist Handbook. Slough: NFER-NELSON; 1989.

Miyake K, Ohmura M, Takashima M, Yamauchi S, Hashimoto Y. Kinder Infant Development Scale: Manual. Tokyo: Hattatsu Kagaku Kenkyu Kyoiku Center; 1989.

Meyer TJ, Miller ML, Metzger RL, Borkovec TD. Development and validation of the Penn State Worry Questionnaire. Behav Res Ther. 1990;28:487–95. doi:10.1016/0005-7967(90)90135-6.

Mullen EM. Mullen scales of early learning. London: Pearson; 1995.

Öner N, Le Compte A. Durumluluk Süreklilik Kaygı Envanteri Elkitabı. Boğaziçi Üniversitesi Yayınları; 1985.

Orthar G, Ben Shahar N. Reading Comprehension Test. Ministry of Education; 1976.

Palisano R, Rosenbaum P, Walter S, Russell D, Wood E, Galuppi B. Development and reliability of a system to classify gross motor function in children with cerebral palsy. Dev Med Child Neurol. 1997;39:214–23. https://doi.org/10.1111/j.1469-8749.1997.tb07414.x.

Paraskevopoulos I. Athina Test: Screening test of learning disabilities, revised edition. Athens; 2011.

Perrin EC, West PD, Culley BS. Is my child normal yet? Correlates of vulnerability. Pediatrics. 1989;83:355–63.

Pianta RC. Child-Parent Relationship Scale (unpublished measure). Virginia: University of Virginia; 1995.

Prinz RJ, Foster S, Kent RN, O'Leary KD. Multivariate assessment of conflict in distressed and nondistressed mother-adolescent dyads. J Appl Behav Anal. 1979;12:691–700. https://doi.org/10.1901/jaba.1979.12-691.

Raven J, Raven JC, Court JH. Raven Manual: Section 3. SPM Manual. Oxford: Oxford Psychologists Press; 2000.

Ravens-Sieberer U, Bullinger M. Assessing healthrelated quality of life in chronically ill children with the German KINDL: First psychometric and content analytical results. Quality of Life Research. 1998;7:399–407.

Reitan RM. Manual for Administration of Neuropsychological Test Batteries for Adults and Children. Neuropsychology Laboratory. 1979.

Rey A. L’examinen psychologique dans les cas d’encephalopathie traumatique. Archives de Psychologie. 1941;28:286–340.

Reynolds CR, Kamphaus RW. Behavior Assessment System for Children. Circle Pines, MN: American Guidance Service; 1992.

Robbroeckx LMH, Wels PMA. Nijmegen questionnaire regarding child-rearing circumstances. Nijmegen: Katholieke Universiteit Nijmegen; 1989.

Rohner RP. Handbook for the Study of Parental Acceptance and Rejection. Connecticut: University of Connecticut; 1999.

Rong Z, Houcan Z. Development of the CDCC Chinese child development scale (3–6 years old). Chin. J. Psychol Sci. 1994;17:137–40.

Roussou A. Manual for the ASEBA School-Age Forms & Profiles (Greek version). Athens: Ellinika Grammata; 2003.

Rust J, Bennun I, Crowe M, Golombok S. The handbook of the Golombok Inventory of Mariatl State. Slough: NFER-Nelson; 1988.

Rust J, Bennun I, Golombok S. The GRIMS: A psychometric instrument for the assessment of marital discord. J Fam Ther. 1990;12:45–57.

Rutter M. A children’s behaviour questionnaire for completion by teachers: Preliminary findings. J Child Psychol Psychiatry. 1967;8:1–11. https://doi.org/10.1111/j.1469-7610.1967.tb02175.x.

Rutter M, Tizard J, Whitmore K. Education, health and behaviour. London: Longman; 1970.

Scheier MF, Carver CS. Optimism, coping, and health: assessment and implications of generalized outcome expectancies. Health Psychol. 1985;4:219–47. https://doi.org/10.1037//0278-6133.4.3.219.

Shani M, Lachman D, Shalem T, Bahat A. Alef-Tav Examination for Diagnosis of Reading and Writing Processes According to National Norms: Theoretical and Practical Manual. Tel Aviv: Yesod Publ; 2006.

Shepherd M, Oppenheim B, Mitchell S. Childhood behavior and mental health. London: University of London Press; 1971.

Smilansky S, Shephatia L. Manual for teachers. Szold Institute; 1976.

Smilkstein G. The Family APGAR: A proposal for family function test and its use by physicians. The Journal of Family Practice. 1978;6:1231–9.

Soenens B, Beyers W, Vansteenkiste M, Sierens E, Luyckx K, Goossens L. The “gross anatomy” of parenting styles in adolescence: three or four dimensions? 18th biennial meeting of the International Society for the Study of Behavioural Development (ISSBD). Ghent; 2004.

Spanier GB. Measuring Dyadic Adjustment: New Scales for Assessing the Quality of Marriage and Similar Dyads. J Marriage Fam. 1976;38:15–28. https://doi.org/10.2307/350547.

Sparrow SS, Balla DA, Cichetti DV. Vineland Adaptive Behaviour Scales (Interview Edition). Circle Pines, MN: American Guidance Services; 1984.

Sparrow SS, Cicchetti DV, Balla DA. Vineland adaptive behavior scales. 2nd ed. Circle Pines, MN: American Guidance Service; 2005.

Spielberger CD. Theory and research on anxiety. In Spielberger CD, editor. Anxiety and behavior. New York: Academic Press; 1966. pp. 3–20.

Spielberger CD. State-Trait Anxiety for Children: Preliminary Manual. California: Consulting Psychologists Press; 1973.

Spielberger CD. The Handbook of the State-Trait Anxiety Inventory. California: Consulting Psychologists Press; 1983.

Spielberger CD, Gorsuch RL, Lushene R. The State-Trait Anxiety Inventory test manual. California: Consulting Psychologists Press; 1970.

Spielberger CD, Gorsuch RL, Lushene R, Vagg PR, Jacobs GA. Manual for the State-Trait Anxiety Inventory, STAI (Form Y) Self-Evaluation Questionnaire. California: Consulting Psychologists Press Inc; 1983.

Stein RE, Riessman CK. The development of an impact-on-family scale: preliminary findings. Med Care. 1980;18:465–72. https://doi.org/10.1097/00005650-198004000-00010.

Straus MA. Measuring intrafamily conflict and violence: The conflict tactics scale (CTS). J Marriage Fam. 1979;41:75–88. https://doi.org/10.2307/351733.

Sutter J, Eyberg S. Sutter-Eyberg Student Behavior Inventory. Lake Magdalene: Psychological Assessment Resources; 1984.

Tavor ST. Speech Tests and Training, Tavor Picture Naming Expressive Vocabulary Test for children age 2 to 8: Manual. 2011.

Tedeschi RG, Calhoun LG. The Posttraumatic Growth Inventory: measuring the positive legacy of trauma. J Trauma Stress. 1996;9:455–71. https://doi.org/10.1007/BF02103658.

The Australian Early Development Census. ABOUT THE AEDC. 2020. https://www.aedc.gov.au/about-the-aedc. Accessed Nov 9 2024.

The Australian Curriculum Assessment and Reporting Aurthority (ACARA). The National Assessment Program for Literacy and Numeracy. 2023. https://nap.edu.au. Accessed Nov 9 2024.

Thomasgard M, Metz WP, Edelbrock C, Shonkoff JP. Parent-child relationship disorders. Part I. Parental overprotection and the development of the Parent Protection Scale. J Dev Behav Pediatr. 1995;16:244–50.

Tiffin J. Purdue Pegboard: Examiner Manual. Chicago: Science Research Associates; 1968.

Van der Ploeg HM. Validatie van de Zelfbeoordelingsvragenlijst (een Nederlandstalige bewerking van de Spielberger State-Trait Anxiety Inventory). Ned Tijdschr Psychol. 1980;35:243–9.

Vander Steene G, Bos A. Wechsler Preschool and Primary Scale of Intelligence. Vlaams-Nederlandse Aanpassing. 2nd ed. Testinstructie Swets Test Publishers; 1997.

Vander Steene G, van Haasen PP, De Bruyn EEJ, Coetsier P, Pijl YL, Poortinga YH, lutje Spelberg HC, Spoelders-Claes R, Stinissen J. Wechsler Intelligence Scale for Children – Revised. Nederlandstalige Uitgave. Leiden: Swets & Zeitlinger; 1986.

Verhulst FC, van der Ende J, Koot HM. Handleiding voor de CBCL/4–18. Rotterdam: Erasmus University; 1996.

Verhulst FC, van der Ende J, Koot HM. Handleiding voor de Teacher’s Report Form (TRF). Sophia Kinderziekenhuis, Afdeling Kinder- en Jeugspsychiatrie. Rotterdam: Erasmus University; 1997a.

Verhulst FC, van der Ende J, Koot HM. Handleiding voor de Youth Self-Report/11–18. Rotterdam: Erasmus University; 1997b.

Verhulst FC, van der Ende J, Koot HM. Handleiding voor de CBCL/4–18. Rotterdam: Sophia Kinderziekenhuis, Erasmus MC; 2000.

Watson D, Clark LA, Tellegen A. Development and validation of brief measures of positive and negative affect: the PANAS scales. J Pers Soc Psychol. 1988;54:1063–70. https://doi.org/10.1037//0022-3514.54.6.1063.

Watson M, Greer S. Development of a questionnaire measure of emotional control. J Psychosom Res. 1983;27:299–305.

Wechsler D. Wechsler Intelligence Scale for Children – Revised: Manual. New York: Psychological Corporation; 1974.

Wechsler D. Wechsler Intelligence Scale for Children – Revised: Hebrew standardization. Jerusalem: Hebrew University Jerusdem; 1976.

Wechsler D. Wechsler Preschool and Primary Scale of Intelligence – Revised Manual. New York: Psychological Corporation, Harcourt Brace Jovanovich Inc; 1989.

Wechsler D. Manual for the Wechsler Preschool and Primary Scale of Intelligence – Revised. New York: Psychological Corporation; 1990.

Wechsler D. Manuel: echelle d’intelligence de Wechsler pour la période préscolaire et primaire. Forme révisée. Paris: ECPA; 1995.

Wechsler D. Wechsler Abbreviated Scale of Intelligence WASI Manual (4th ed.). London: Pearson/PsychCorp; 1999a.

Wechsler D. Manual for the Wechsler Preschool and Primary Scale of Intelligence – Revised. Psykologiförlaget; 1999b.

WHOQUAL Group. Development of the World Health Organization WHOQOL-BREF Quality of Life Assessment. Psychol Med. 1998;28:551–8. https://doi.org/10.1017/S0033291798006667.

Zimmer R, Volkamer M. MOT 4–6, Motoriktest für 4–6jährige Kinder. Göttingen: Hogrefe; 1987.

Zung WW. A Self-Rating Depression Scale. Arch Gen Psychiatry. 1965;12:63–70. https://doi.org/10.1001/archpsyc.1965.01720310065008.
